# Humans versus models: a comparative assessment of ecosystem services models and stakeholders’ perceptions

**DOI:** 10.1038/s41598-024-76600-w

**Published:** 2024-10-29

**Authors:** João David, Pedro Cabral, Felipe S. Campos

**Affiliations:** 1 Humboldt-Universität zu Berlin, Geography Department, Landscape Ecology Lab, Rudower Chaussee 16, 12489 Berlin, Germany; 2 School of Remote Sensing and Geomatics Engineering, Nanjing University of Information Science and Technology, Nanjing, 210044 China; 3 NOVA Information Management School (NOVA IMS), Universidade Nova de Lisboa, Campus de Campolide, 1070-312 Lisboa, Portugal; 4 Universitat Autònoma de Barcelona, 08193 Cerdanyola del Vallès, Catalunya Spain; 5 Centre de Recerca Ecològica i Aplicacions Forestals (CREAF), 08193 Cerdanyola del Vallès, Catalunya Spain

**Keywords:** Land use changes, Ecosystem services indicators, Landscape planning, Stakeholders’ perception, Sustainable management, Ecosystem services, Ecological modelling, Environmental impact, Sustainability

## Abstract

Mapping the production of Ecosystem Services (ES) is imperative for sustainable ecosystem management. Likewise, incorporating expert knowledge enhances ES research. Here, we calculate eight multi-temporal ES indicators for mainland Portugal using a spatial modelling approach. These indicators are then integrated into the novel ASEBIO index—Assessment of Ecosystem Services and Biodiversity—which depicts a combined ES potential based on CORINE Land Cover, using a multi-criteria evaluation method with weights defined by stakeholders through an Analytical Hierarchy Process (AHP). Outputs from the modelling show how ES have changed in Portugal in relation to land use changes, including trade-offs between 1990 and 2018. The composed ASEBIO index is compared against the stakeholders’ valuation of ES potential for the year 2018. The results reveal a significant mismatch between the ES potential perceived by stakeholders and the models, with stakeholder estimates being 32.8% higher on average. All the selected ES were overestimated by the stakeholders. Drought regulation and erosion prevention have the highest contrasts, while water purification, food production and recreation are the most closely aligned among both approaches. Providing the first national overview about the status of multiple ES over a 28 year-period, our findings highlight potential disparities between data-driven and stakeholder-based evaluations. Therefore, we suggest the need for integrative strategies that consider scientific models with expert knowledge for more effective ES assessments and land-use planning. This approach could help bridge the gap between data-driven models and human perspectives, resulting in more balanced and inclusive decision-making.

## Introduction

Ecosystem services (ES), i.e. the benefits that ecosystems provide to humans, are crucial for sustaining well-being and the global economy^1^. However, these services are under increasing threat from anthropogenic pressure and land cover changes, leading to significant losses in biodiversity and ecosystem functionality^2^. Mapping and assessing ES is therefore crucial to support decision making and to monitor the progress towards relevant policies such as the United Nations Sustainable Development Goals (SDG)^3^.

Trends in ES research include social-ecological integration, landscape functioning, and the monitoring and measurement of ES over space and time^4–6^. Researchers acknowledge that involving stakeholders from various sectors of society to collect their perceptions is necessary to ensure a comprehensive understanding and sustainable management of ES, even if stakeholders may hold differing views regarding the same ES^7,8^. It is also crucial to understand and communicate the effects of land cover changes on different ES, as human impacts on land use may decrease ES potential^9,10^. Nevertheless, data on ES is rarely included in conservation planning^11^. However, ES monitoring could function as an integrative tool to shift the paradigm and inform land-use management with different approaches^12^.

Distinct methods to study ecosystems and their services are discussed in the literature^13,14^. Understanding trade-offs between multiple ES, rather than focusing on a single service, is fundamental for leveraging ES knowledge^15^. Spatiotemporal assessments are also key to identifying the dynamics and changes of ES through space and time, and for designing monitoring programs for future ES^16,17^. Moreover, meaningful data and indicators to quantify ES are needed^18^. Geographic Information Systems are essential tools for the spatial assessment of ES, enabling the visualization and analysis of these services. For instance, the integration of ES in European Union policy relies on spatial information^19^. Despite the availability of many decision-support tools^20,21^, ES synergies can be measured with simple models^22^. One such example is the InVEST software (Integrated Valuation of Ecosystem Services and Tradeoffs)^23^, a spatial modelling tool that estimates and raises awareness of various ecosystems^24^. InVEST is widely used for planning and research applications^25^.

In Portugal, ES applications are conducted as case studies, with papers mostly targeting local^26–28^ or regional study areas^29–31^, and some authors engaging with stakeholders^32–34^. It is only recently that single biophysical and economic ES assessments on national scales were conducted (e.g.^35–37^). While national and global ES studies are common^38^, limited attention has been given to assessing multiple ES with a spatiotemporal perspective and integrating these assessments into a comprehensive ES index. Besides, although ES studies are well-documented in the literature, there is a gap in comparing and evaluating model-based ES with stakeholder perceptions across large spatial scales. Most studies focus either on biophysical models to quantify ES or on involving stakeholders to capture their knowledge and perceptions, but few have integrated these approaches to investigate how scientific models align with or differ from human perceptions. This is particularly relevant given the increasing reliance on models for policymaking, which may not always reflect stakeholder values and perceptions.

Considering the ecological challenges that countries deal with, this paper aims to assess and monitor ES in mainland Portugal and to understand the impacts of land cover changes, with three specific objectives in mind: i) to study the spatiotemporal changes of ES in mainland Portugal between 1990 and 2018; ii) to create an ES index that integrates multiple ES indicators; and iii) to compare the results of ES indicators produced by a spatial modelling approach against the potential of ES perceived by stakeholders. To achieve these goals, we first estimated eight distinct ES indicators for the reference years of 1990, 2000, 2006, 2012 and 2018, using a spatial modelling approach supported by land cover cartography. We then developed the assessment of ecosystem services and biodiversity (ASEBIO) index, which combines ES data models with stakeholder-defined weights, assigned through a multi-criteria evaluation method to reflect the relative importance of each service’s supply potential^37^. Finally, we quantified the differences between the ASEBIO index results and a matrix-based methodology reflecting stakeholders’ ES perceptions. In doing so, this research sheds light on the potential disparities between spatial modelling assessments and human knowledge, which should be an impact for sustainable land-use planning. Our findings could be useful for policymakers aiming to combine scientific data with stakeholder input in ecosystem management. The insights gained here could help with more balanced and inclusive approaches to land-use planning, ensuring that both data-driven and local perspectives are sufficiently considered. Furthermore, this is the first attempt to compare national-scale ES assessments based on modelling methodologies with stakeholder-perceived ES potential.

## Results

### Ecosystem services indicators: temporal changes from spatial models

**Fig. 1:**
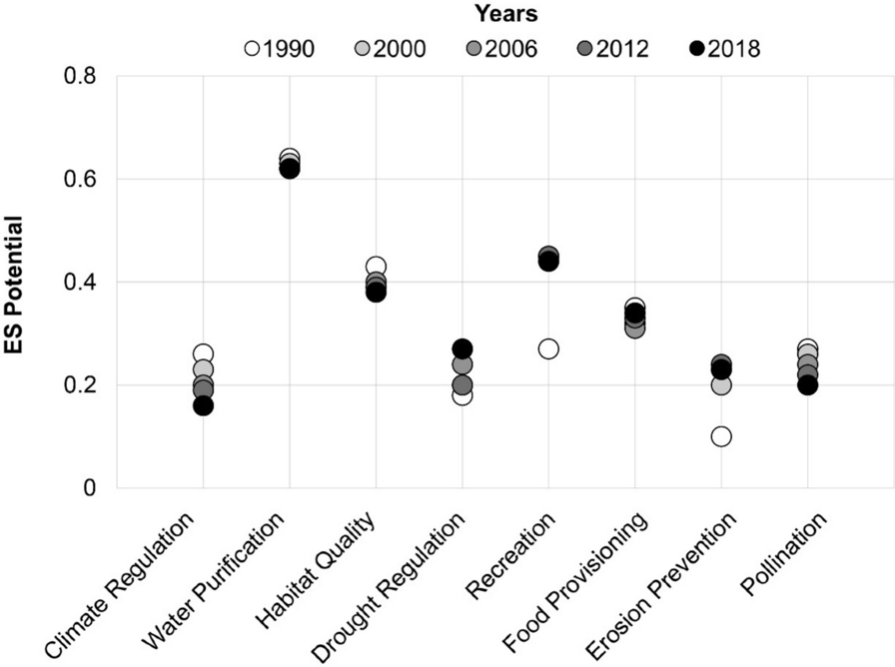
ES potential indicator evolution between 1990 and 2018 based on land cover classes. 0 = No potential, 1 = Maximum potential.

The analysis of land cover changes from 1990 to 2018 showed significant shifts in ES indicators (Fig. 1), which mean values varying across all periods (F = 1.584, *P* < 0.001). Erosion prevention had a wide range of values but very low potential in 1990. Water purification on the other hand consistently showed high potential throughout the years. Climate regulation potential declined, while drought regulation, erosion prevention, and recreation all improved. Habitat quality, food provisioning, and pollination remained mostly stable, with slight declines. Overall, habitat quality and recreation showed twice the potential compared to climate regulation, drought regulation, erosion prevention, and pollination, whereas water purification showed triple the potential.

**Fig. 2:**
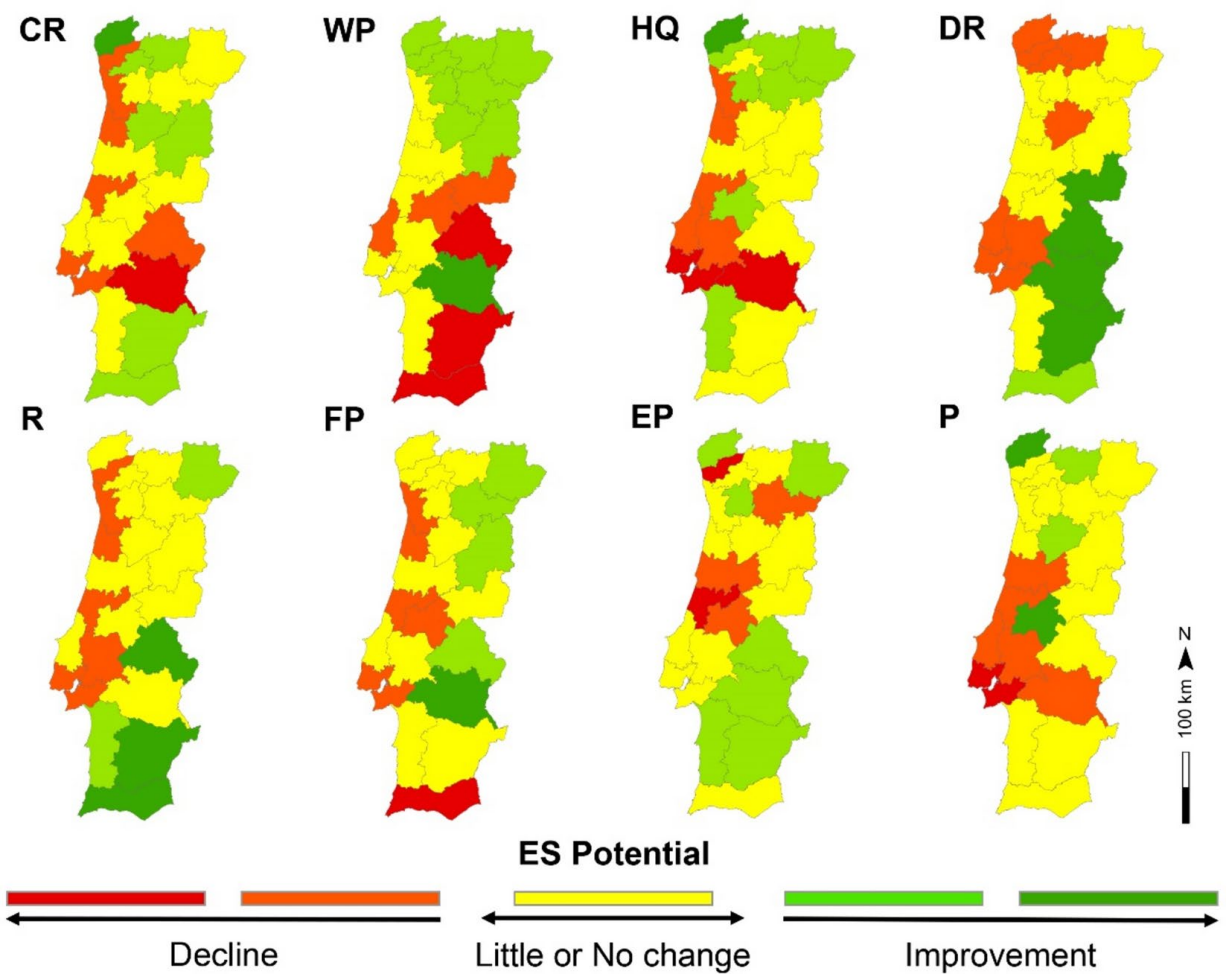
ES potential indicator changes between 1990 and 2018 by NUTS-3 in mainland Portugal (*CR* climate regulation; *WP* water purification; *HQ* habitat quality; *DR* drought regulation; *R* recreation; *FP* food provisioning; *EP* erosion prevention; *P* pollination). The administrative boundaries are illustrated in Fig. S1 (supplementary information). The maps were generated using ArcGIS Pro software version 3.2 (ESRI: https://www.esri.com/en-us/arcgis/products/arcgis-pro/overview).

A set of eight maps illustrate changes to ES potential (%) between 1990 and 2018 across NUTS-3 regions, revealing spatial distribution differences for each indicator (Fig. 2). The scale of analyses ensures an accurate visualization of temporal changes across the defined geographic space, aiding in regional and ES-specific comparisons. Climate regulation declined notably (darker red) in Alentejo Central and improved (darker green) in Alto Minho, whereas water purification improved in 10 out of 23 regions, mostly in the north, while the interior and southern regions experienced declines. Habitat quality increased in the north but declined in Lisbon metropolitan area and Alentejo Central. Drought regulation showed the largest improvement, especially in central and southern regions, but declined in 8 regions. Recreation was found to improve in the Algarve and the interior but declined in coastal areas. Food provisioning decreased in the Algarve but improved in many interior regions. Erosion prevention decreased in Cávado and its region. Pollination potential mostly remained unchanged, with declines in some contiguous regions.

Lisbon and Porto metropolitan areas did not show improvements in the selected ES. Lisbon had declines in six ES indicators, with only water purification and erosion prevention not declining. Porto on the other hand, experienced declines in four indicators, with minor changes in drought regulation and pollination.

### The ASEBIO index from 1990 until 2018

**Fig. 3:**
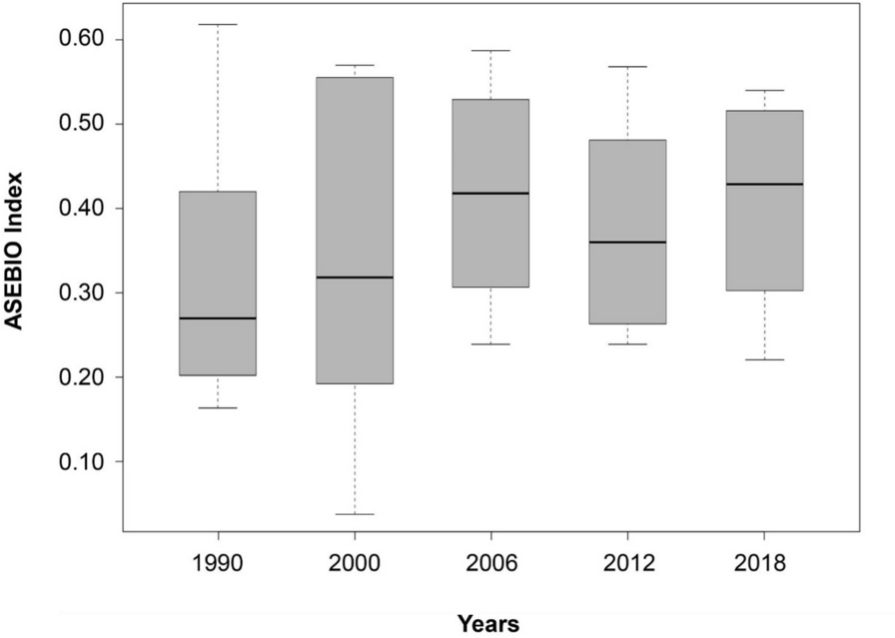
The ASEBIO index for mainland Portugal between 1990 and 2018. Boxplots show the median, upper and lower quartiles, and minimum and maximum observed values.

The ASEBIO index depicted the overall combined ES potential based on CORINE Land Cover, adopting a multi-criteria evaluation method with weights defined by stakeholders through an Analytical Hierarchy Process (AHP). From 1990 to 2018, significant differences were observed in the averages of ES distribution levels (F = 1.632, *P* = 0.029; Fig. 3). The index median values were lowest in 1990 (0.27) and highest in 2018 (0.43). Median values increased from 1990 to 2006 (0.27 to 0.41), decreased between 2006 and 2012 (0.41 to 0.35), and rose again in 2018 (0.43). The maximum index value was in 1990 (0.62), whereas the minimum index value was in 2000 (0.02). The largest interquartile range was observed for the year 2000, with similar ranges observed in 1990, 2006, 2012, and 2018. This means that the overall distribution of values of the 8 ES indicators was more similar among themselves over these years. Overall, the ASEBIO index values for the timeline remained relatively stable (0.33–0.35).

Water purification was the indicator that contributed the most to the ASEBIO index in all years. In 1990, habitat quality was the next highest contributor, while erosion prevention contributed the least. By the year 2000, recreation doubled its potential and became the highest contributor, with erosion prevention still being the lowest. In 2006, 2012, and 2018, recreation remained a major contributor below water purification, while climate regulation replaced erosion prevention as the least contributing ES.

### Land cover contribution to ASEBIO index estimates

**Fig. 4:**
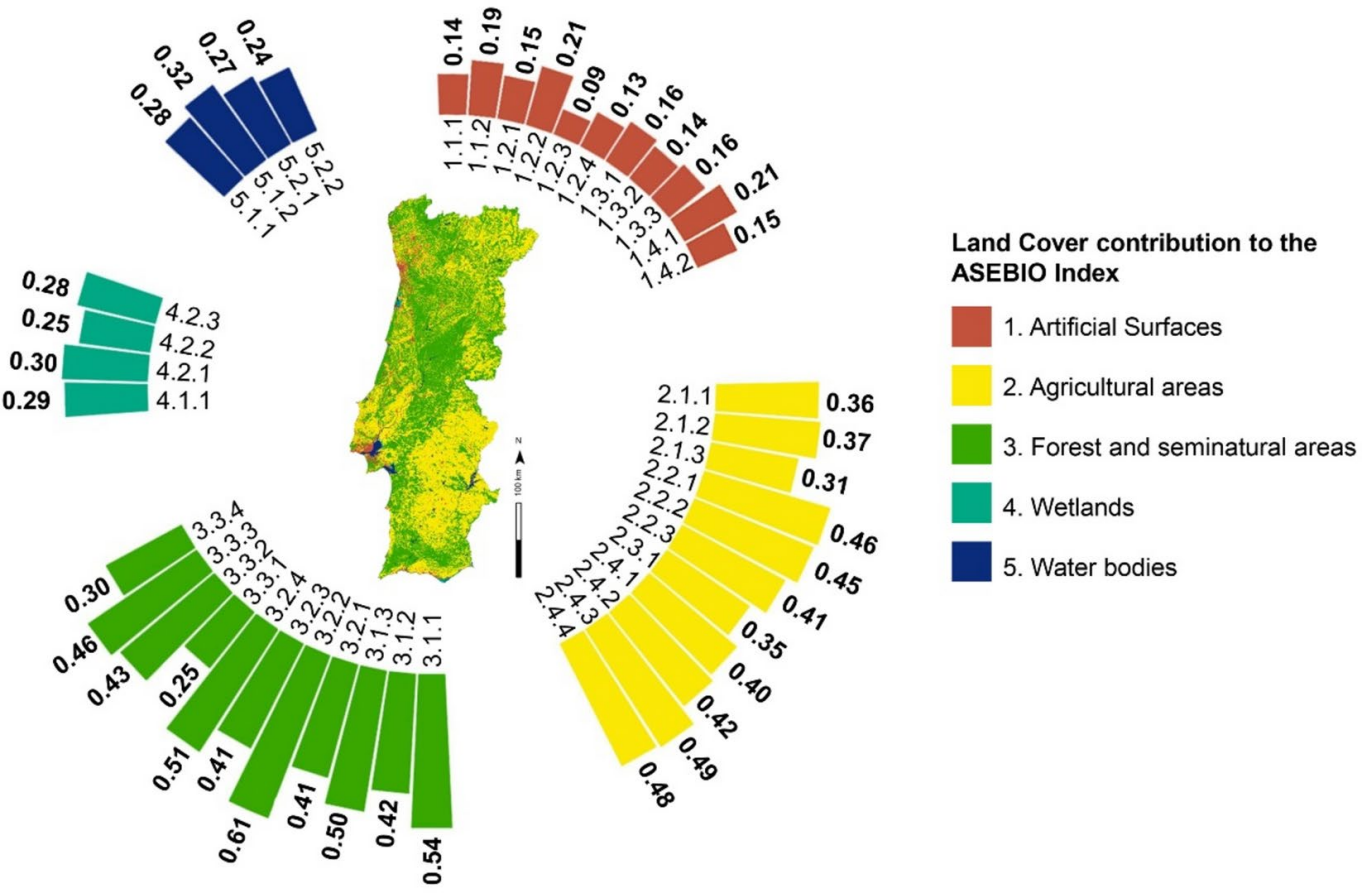
Land cover contribution to the ASEBIO index in 2018. Average values by land cover classes are represented according to the CORINE level 3 nomenclature (see supplementary information). The map was generated using ArcGIS Pro software version 3.2 (ESRI: https://www.esri.com/en-us/arcgis/products/arcgis-pro/overview).

Considering the spatial heterogeneity of mainland Portugal’s landscape, each land cover class had a different contribution to the ASEBIO index for the year 2018 in terms of relative importance and weighting (Fig. 4). Port areas (1.2.3) contributed the least to the index, whereas from the artificial surfaces category, road and rail networks and associated land (1.2.2) and green urban areas (1.4.1) contributed the most. From the agricultural areas, rice fields (2.1.3) contributed less compared to other classes. Land primarily used for agriculture with significant natural vegetation (2.4.3) and agro-forestry areas (2.4.4) had a substantial influence, which was greater than most forest classes. Wetlands and water bodies contributed almost equally. On average, forest and seminatural areas were the main contributors to the ASEBIO index, with moors and heathland (3.2.2) having the highest values.

### The ASEBIO index compared to stakeholders’ perception for 2018

**Fig. 5:**
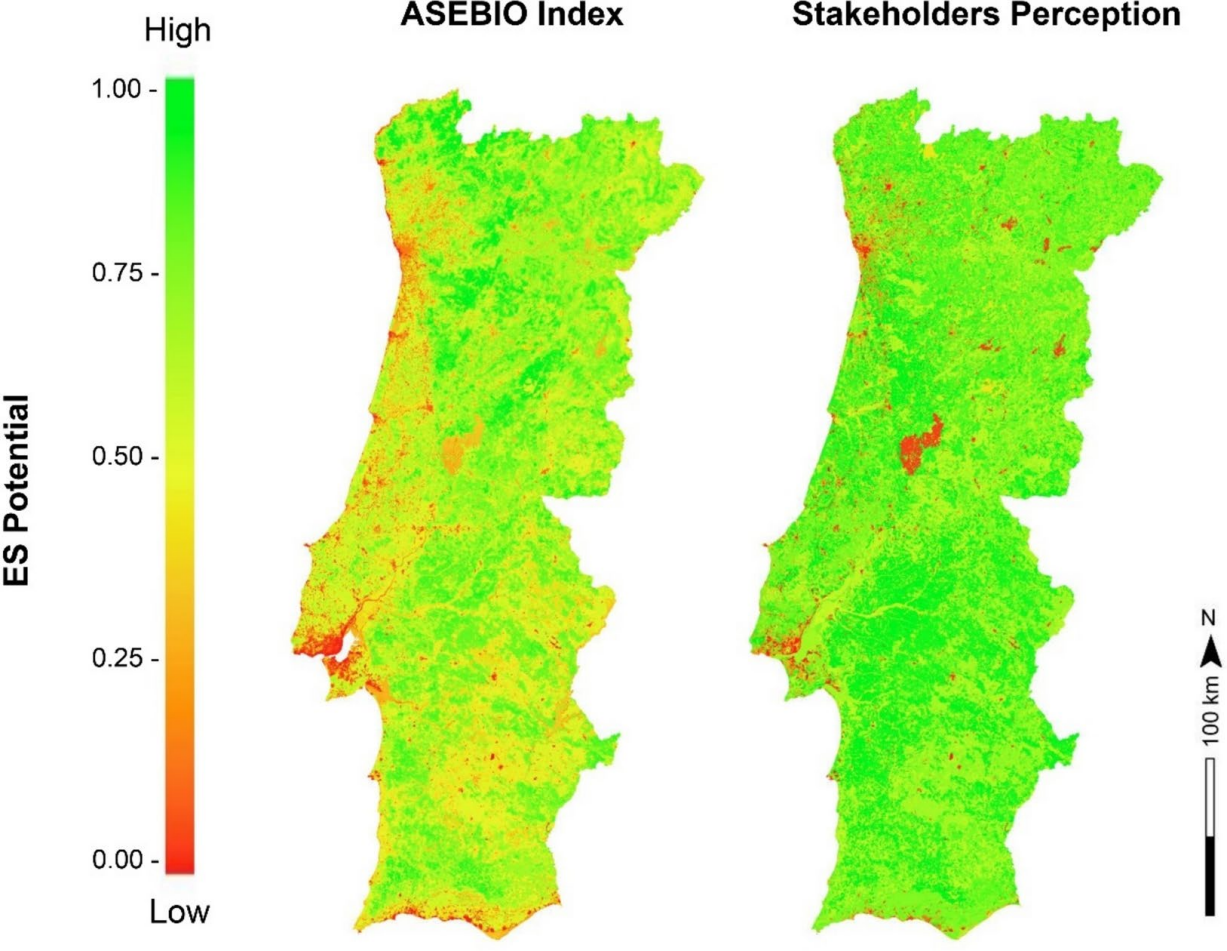
Spatial distribution differences between the actual overall ES potential for 2018 obtained by the ASEBIO index (left) and the ES perceived by stakeholders’ (right) in mainland Portugal. The maps were generated using ArcGIS Pro software version 3.2 (ESRI: https://www.esri.com/en-us/arcgis/products/arcgis-pro/overview).

Maps in Fig. 5 revealed the spatial distribution differences of ES potential in mainland Portugal considering landscape attributes. The map on the left displayed the ASEBIO index, summarizing the eight ES indicators obtained through spatial modelling. The map on the right map showed the average ES potential for each land cover class according to stakeholders’ perceptions. Both methods incorporated stakeholder-determined weights of importance. When adopting an equal colour scale from low (red) to high (green) to show ES potential, it was noticeable that the stakeholders’ perception map appears to be greener than the ASEBIO Index map. This indicated that stakeholders generally value ES potential higher than the modelling approach. In despite of this however, spatial patterns for both maps were relatively similar, showing a moderate positive correlation (r = 0.527, *P* < 0.001).

**Fig. 6:**
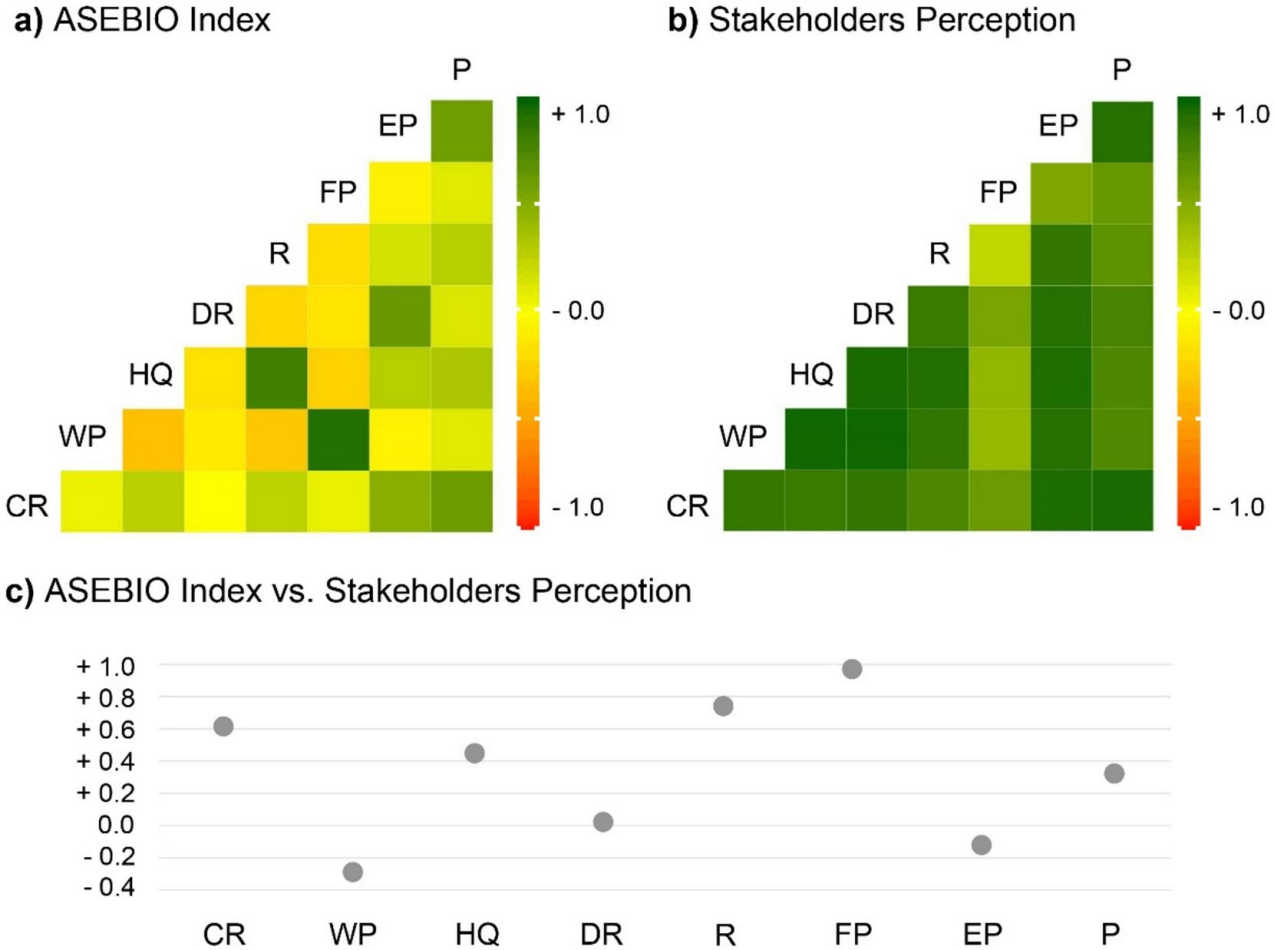
ES potential correlation of models and stakeholders based on land cover (*CR* climate regulation; *WP* water purification; *HQ* habitat quality; *DR* drought regulation; *R* recreation; *FP* food provisioning; *EP* erosion prevention; *P* Pollination).

A correlation matrix identified the strength and type of relationship among the ES models from the ASEBIO index and the stakeholders’ approaches, based on the land cover contribution (Fig. 6a,b). The correlations within the ASEBIO index were generally lower than those perceived by stakeholders. Within the ASEBIO index, habitat quality and recreation, as well as water purification and food provision, were highly positively correlated. As for the stakeholders’ perceptions, all ES were positively correlated, mostly exhibiting very high correlations. The association between the ASEBIO index and the stakeholders’ perception maps showed distinct spatial correlations (Fig. 6c). Drought regulation and erosion prevention models exhibited no correlation with stakeholders’ perceptions, meaning that there was no spatial relationship between the variables. In contrast, water purification was negatively correlated between stakeholders’ valuations and the models’ predictions, showing a geographical mismatch among raster values. Conversely, recreation and food provisioning showed high to very high correlations, suggesting that the raster values in both approaches had a similar spatial pattern.

Comparing the differences between both approaches using the statistical mean across the entire study area, results revealed that the overall ES potential assessed by stakeholders was 32.8% higher than the ASEBIO index estimates. For each ES indicator, stakeholders’ perceptions were always higher than the spatial models integrated within the ASEBIO index (Table 1). Drought regulation, erosion prevention, and pollination showed the greatest differences, while water purification showed the most similar values between both methods, followed by food provisioning and recreation. *T*-test results indicated a significant statistical difference between the means obtained by the ES models and stakeholders’ approaches (t = 3.151, *P* = 0.002).

**Table 1:** Comparison between the ASEBIO index average values and stakeholders’ ES average potential in mainland Portugal for 2018.

ES indicator	CR	WP	HQ	DR	R	FP	EP	P
Average for models	0.43	0.66	0.46	0.22	0.49	0.48	0.16	0.42
Average for stakeholders	0.73	0.68	0.73	0.70	0.66	0.59	0.67	0.77
Relative difference	0.30	0.02	0.26	0.56	0.17	0.11	0.51	0.35

*CR* climate regulation, *WP* water purification, *HQ* habitat quality, *DR* drought regulation, *R* recreation, *FP* food provisioning, *EP* erosion prevention, *P* pollination.

## Discussion

### Insights from ecosystem services assessments

Our temporal assessment of ES indicators in Portugal shows consistent results with few variations across a 28-year span. Climate regulation potential progressively declined from 1990 to 2018, possibly due to reduced carbon sequestration and extensive wildfires caused by the abandonment of rural areas and climate change^39^. These results align with the findings proposed by Pacheco^40^ on forest and fire management’s impact on climate regulation. Habitat quality follows a similar pattern to climate regulation, decreasing through time, which can be explained by the increase of land use activities reducing habitat suitability. We observed how climate and habitat models tend to have similar performances, suggesting that biodiversity conservation can be enhanced from better climate regulation services, as proposed by Campos et al.^41^. Recreation was the indicator that improved the most from 1990 to 2018, with twice as many high-quality areas for recreational services. The emerging attention given to protected areas coupled with the accessibility of nature-based recreation to people provides evidence of such changes and is consistent with European trends^42^. Drought regulation also enhanced its potential over time. According to Parente et al.^43^, the variability of drought regulation in Portugal is influenced by water management strategies and a set of climate factors, including precipitation and temperature patterns. Besides these factors, our model suggests that this variability may also be influenced by land cover changes. Results suggest that pollution avoided by nitrogen retention is highly retained on the Portuguese landscape (i.e., nutrients are mostly out from streams, ensuring a good water quality for drinking). However, at wider scales, this model can differ with the sources of nitrogen loads that drive the variability in water quality, and their relative differences between regions^44^. An improvement in the erosion service since 1990 could be related to an increase in forest vegetation over recent decades^45^. Food provisioning and pollination services showed low variation, likely shaped by consistent crop yields. This stability could result from ES trade-offs, as observed in related studies^44,46^. The ASEBIO index revealed increasing ES potential from 1990 to 2018, with a moderate decrease from 2006 to 2012, which could be associated with wildfires and the loss of forests^47,48^. The index suggests robust and balanced ES values over time. By combining ES indicators into an index that reflect stakeholders’ priorities, this provides a comprehensive overview of regional ES status.

Our findings support the results of Petersen et al.^49^ in that ecosystem extents have almost remained stable in Europe since 2000, except urban systems, which are expanding. Most of the variations in the assessed ES relate to existing processes of land use changes. Land cover patterns, aligned with biophysical features of ES models, endorse our achievements. For this purpose, we appraised either the biophysical models or the land cover ES potential. This is particularly relevant for understanding which environments contributed more or less to the eight assessed ES indicators in 2018. In fact, land use intensity impacts on ES are only weakly perceived^50^. We suggest a balanced contribution among the major land cover categories for providing multiple ES. One exception to this are artificial surfaces, with little potential. However, it is important to preserve green infrastructures in artificial surfaces for urban ES^51^.

It is also noteworthy that the country’s two metropolitan areas, Porto, and Lisbon, showed no improvements in ES potential over the analysed period, illustrating a long-term declining trend. This interpretation supports the findings of Nicolau et al.^52^, who revealed that the widespread expansion of Portugal’s urban areas and a decrease of the population in those areas, is causing an inefficient land use. This topic is especially relevant since urban areas are hot spots for driving environmental change on multiple scales^53^, and such changes have an impact on ES capacity to society^54^. Therefore, we recommend planners to focus on these results as restoring urban ES could reduce the cities ecological footprint, avoid the loss of economic costs, and improve their resilience^55^.

Our study addresses the lack of ES information in Europe^56^. Therefore, we wish to propose our results for planning interventions. Related hands-on data are provided through an open WebGIS (https://asebio.novaims.unl.pt/#webgis), which enables a spatially explicit exploration of ES scenarios on different spatial and temporal scales. Within the platform, users can explore and visualise all the modelled ES from this research. Webmaps display the ES potential with a 100 m spatial resolution. We highlight the importance of this interactive instrument to local, regional, and national planners and decision-makers.

### Implications for ecosystem services assessments

We conducted empirical research to measure and compare ES data-based models with stakeholders’ ES perception. Therefore, our study considered: a) the potential of ES through a spatial modelling; and b) the potential of ES perceived by stakeholders given the ability of land cover classes to provide ES. When comparing data-based modelling with stakeholders’ perceptions, we found that overall, ES values can be overestimated by more than 30%. Taking into account the Portuguese land-use dynamics, our results found stakeholders overestimating all individual ES indicators, with the most excessive values related to drought regulation, likely reflecting the high importance stakeholders place on drought mitigation in a country like Portugal, which is vulnerable to extreme climate events. Water purification, on the other hand, showed almost no differences between the average values of both approaches. This might be because water quality issues are more observable and directly experienced by stakeholders, such as through drinking water sources or related pollution. Besides, while less pronounced, the recreation and the food production services also had similarities in the average values of both applications. This is likely because both services are tangible and easily observed. Recreation involves visible landscapes and accessible locations, while food production is directly tied to observable agricultural activities. The discrepancy between model outputs and stakeholder perceptions underscores the importance of integrating both approaches for ES assessments.

In a nutshell, the ASEBIO index and the stakeholders’ perception maps deliver identical visualization patterns. Areas mapped as low potential in one map, are also areas of low potential in the other map. Yet, stakeholders also perceived certain areas to deliver high potential, which are not considered as high potential by the quantitative modelling assessments. Therefore, what differs is the intensity of the provision potential delivered by the ES models (somewhat lower) and the stakeholders’ perspectives (somewhat higher). This reveals that stakeholders are able to locate low ES potential regions, but also foreseen higher potential in other regions, which is not confirmed by the models. Despite some differences in the average potential for each ES, the spatial patterns between model-based and stakeholder maps remain relatively similar. Although stakeholders overestimate certain values, their general understanding of ES distribution is still in line with the model outputs. In addition, trade-offs within the ASEBIO index models are inherent to the land cover capacity for contributing to ES.

In line with the discussed maps, the high correlation among the ES assessed by stakeholders demonstrates how participants expect land cover classes to deliver a higher potential for ES provision, rather than the actual provisioning potential. Hence, we find stakeholders attributing higher values and priority to all ES potential, whereas in the model, we find those same ecosystems to have considerably less potential to provide services. Understanding these mismatches can avoid misleading landscape planning and support the interplay between ES supply and demand^44,57^. We also presented the trade-offs between the two methods, revealing which ES could be (dis)similar in their assessment. The food provisioning indicator is most likely to get identical results for both approaches. Recreation and climate regulation are the other strongly correlated models, with spatial models or stakeholders’ assessments presumably producing similar outcomes. Water purification provides a weak negative relationship, with lower potential values in some areas of this model representing higher potential values for ES stakeholders’ maps. Non-existent trade-offs are found for drought regulation and erosion prevention. Despite the relative difference in the average values of the ASEBIO index and stakeholders’ perceptions, the spatial autocorrelation may vary across the landscape. 

## Limitations and future research

In spite of the implications of our findings for ES sustainability, it is useful to point out uncertainties, particularly those concerning data quality. Our analyses consider both data-based models and stakeholders’ perspectives, so all the assumptions underlying computer-based decision support tools and expert-based opinions may influence our results^58^. The InVEST model limitations are well documented in the software user guidelines^23^. The obtained results represent the spatial distribution difference between the actual ES calculated using computer models and the ES perceived by the stakeholders. The former is a continuous variable with its own physical meaning, whereas the latter is a discrete variable that represents the opinions of the stakeholders. However, we still found this comparison useful to understand the impact of using each approach on the results and to measure the relative perceptions of stakeholders regarding ES assessments. Although both ES approaches can be independently applied, they both rely on the low precision detailed, coarse spatial resolution, and generalized thematic classification of the CORINE Land Cover maps, which tend to oversimplify ES variation across heterogeneous landscapes^59^.

In participatory approaches for spatial planning, stakeholders are expected to have different perspectives depending on their specific interests in each service in the landscape^37,60^. This leads to a fundamental issue with the stakeholders’ assessments: some participants will base their perceptions on personal beliefs and not on data or processing contexts^57^. Disciplinary paradigms may also reflect final ES scoring^61^. For assessment purposes, we recommend grouping stakeholders to work in their ES of expertise, promoting capacity-building and integrative knowledge. Whenever possible, workshop leaders should seek to deliver exploratory ES maps to assist with stakeholders’ level of knowledge about ES valuation prior to their assessments. A variability and confidence analysis should be envisaged in future steps of this research to improve the consistency of the results^62^.

We assume that combining ES modelling and stakeholders’ methodologies in an integrative way is useful for supporting land-use policies at the national level. Nevertheless, we acknowledge that the ASEBIO index may depend on the study area, as it is made up of specific indicators, values, and weights. Different ES will show a different relevance in different regions. Applying this index to a different geographical context may therefore require further efforts, such as the assignment of weights by a new group of stakeholders. Moreover, the ES assessed in each case will depend on data availability and the specific ES being assessed, which must adapt the index into their needs. Further ES studies should explore how different stakeholder groups perceive and prioritize ES bundles, using diverse modelling approaches and human perspectives to improve real-world management strategies^57^. Therefore, integrating ES data-driven models into decision-making can bridge the gap between scientific understanding and practical application, enabling better environmental and socio-economic outcomes through informed policy and land-use planning^63^.

The geographic study area of our research may explain disparities when comparing both modelling and stakeholders’ approaches. Targeting the whole country as a study area creates a general assumption on ES valuation by stakeholders (who overestimate) and by data-driven approaches (which generalize), potentially bringing some misleading results, especially at a smaller scale. As both methods deliver a distinctive overview about the ES potential for the country, we recommend that land planners choose them in accordance with their goal, and if possible, combine and compare their implications for decision-making processes.

Future research in this area would also benefit the land-use planners and decision-makers in improving ES outcomes for both people and nature^64^. The calibration of the model’s parameters could be discussed with a group of experts on the related ecosystem instead of a general research appraisal. Additional studies using remote sensing data associated with ES dynamics could also be applied from sophisticated sensors^65^. Likewise, robust techniques and different assessment methods are feasible to develop; machine learning capabilities and ES big data leverage ES research at a novel stage^66^.

The main drivers for ES changes analysed in this study are directly related with land use and land cover activities, as the modelling approach is based on CORINE. The application of such datasets increases the international replicability of the study, whereas future research may make use of the forthcoming periodically updated CORINE maps. Current results may be further explored to find spatial hotspots and their relationships with ecologically protected and degraded areas. The extension of this work by projecting different policy scenarios affecting land cover change is a key opportunity to mitigate environmental damages, and to understand the influence of unsustainable land use practices for ES potential.

## Conclusion

This study provides a national-scale assessment of ES in mainland Portugal over a 28-year period, integrating both model-based outputs and stakeholder perceptions. By developing the ASEBIO index, we combined eight ES indicators produced through a spatial modelling approach, allowing for an understanding of the trade-offs in ES provision across varying land cover types. Our results indicate that while the ASEBIO index demonstrates a moderate and balanced evolution of ES potential between 1990 and 2018, considered discrepancies arise when comparing spatial models with stakeholders’ perceptions. Stakeholders overestimated ES by an average of 32.8%, with drought regulation and erosion prevention showing the largest gaps, while water purification, food production and recreation displayed the closest alignments. This mismatch between scientific models and stakeholders’ perceptions underscores the need for integrating human knowledge in data-driven assessments to achieve more inclusive and effective planning strategies. While ES models provide data-driven insights, stakeholders input captures broader social, cultural, and environmental management dimensions of ES, which are often overlooked in purely quantitative models. The ability to incorporate both methodologies in land-use decisions represents a critical step forward in ensuring that planning processes are more reflective of ecological realities and societal needs. In that sense, we provide a scalable methodology for combining different ES, besides offering a framework for comparing models vs human perspectives. While not solving the non-validation limitation in clarifying which approach would better quantify ES indicators, this research contributes with relevant insights into the impact of using each defined approach, providing a direction for future ES research applications. For practical implications in terms of ES management, we share our results interactively through an open WebGIS (https://asebio.novaims.unl.pt/#webgis), allowing practitioners to explore different spatial patterns of multiple ES indicators.

## Methods

### Study area

The research focused on mainland Portugal (Fig. S1, supplementary information). Located in the Iberian Peninsula in southwestern Europe, Portugal shares borders to the north and the east with Spain and to the west and the south with the Atlantic Ocean. It encompasses 89,015 km^2^ and has a population of 10,344,802 inhabitants^67^. The country is geographically divided into north–south (physical aspects) and interior-coastal (population features). The North is mountainous, whereas the South is flat. Rainfall is more prevalent in the North than in the South, which is a region with desertification patterns^68^. Extreme precipitation events and intense dry months are common^69^. In general, agricultural areas are more prevalent the interior and the south, whereas forests dominate the centre. The majority of the population live in littoral regions, and most of the interior communities are predominantly rural. According to CORINE Land Cover (the most appropriate source of land use information on a European level)^70^, agricultural areas account for 47.8% of the entire country, whereas forests and semi-natural areas account for 46.5%, artificial surfaces for 3.8%, water bodies for 1.5%, and wetlands for 0.3%^48^.

### Research framework

**Fig. 7:**
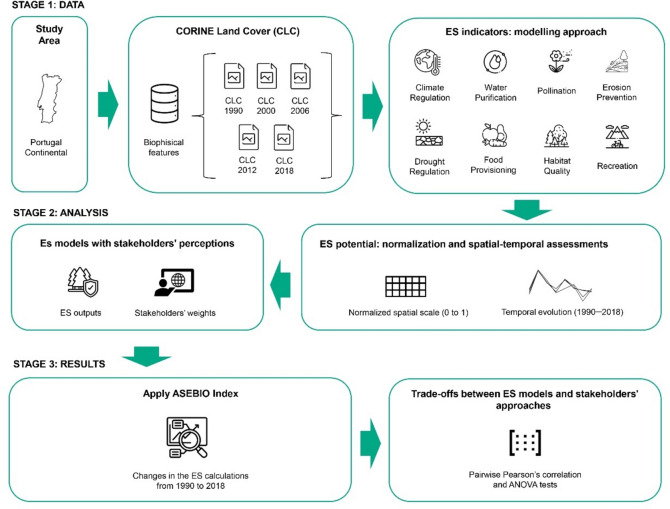
Research framework.

The research framework for this study is structured around a combination of spatial modelling and stakeholder input to assess ES potential in the study area. The flowchart (Fig. 7) illustrates three main stages that summarize the investigation (i.e. data, analysis and results). The first stage focused on the collection and processing the input data and modelling the selected ES. Food provisioning and recreation were calculated using ArcGIS Pro software version 3.2 (ESRI: https://www.esri.com/en-us/arcgis/products/arcgis-pro/overview), whereas all the other indicators were modelled using the InVEST software (version 3.10.2)^23^. In the second stage, the raw outputs for each ES were normalized and analysed, generating both spatiotemporal cartographic and statistical outputs from the models. We also mapped and analysed ES perceived by stakeholders for each selected ES and for a combined ES map which integrates all ES. The final stage involved results developing the integrated model-based ASEBIO index and comparing it with stakeholders’ perceptions of ES potential. The following sections offer a comprehensive explanation of the methodological procedures.

### Ecosystem services indicators: spatial modelling

The research team identified eight ES indicators that are applicable for the study area. The preselection considered the availability of open datasets for ES calculations using modelling tools with the support of land cover maps. Furthermore, the selection followed the same set of ES indicators selected by national stakeholders for informing decision-making processes^37^. These included: 1. Climate Regulation; 2. Water Purification; 3. Habitat Quality; 4. Drought Regulation; 5. Recreation; 6. Food Provisioning; 7. Erosion Prevention; 8. Pollination. The core data common to each indicator is from CORINE for the different time periods of 1990, 2000, 2006, 2012, and 2018^48^. The latest version, CORINE 2018, is a high-quality product for the distribution of ES^71^. Serving as a reference open-source dataset for European countries^72^, CORINE provided a standard nomenclature for a baseline comparison to determine landscape characteristics and ecosystem changes in multiple land contexts within different temporal scales^73^. Raw values from the ES models were harmonized for ease of comparison. Higher values mean a higher potential for a single ES indicator, described in the following subsections. In this context, *potential* refers to the capacity of an ecosystem to provide ES^74^. Cartographic outputs from the modelling stage were also aggregated from a 100 m pixel resolution to provide estimates by NUTS-3 level^75^. This geographic operation has the advantage of enabling comparisons between different regions and being able to assess trends across the study area. Data parameters for each ES modelling approach is found in the supplementary information. The rationale for the calculation of each ES indicator is described in the supplementary information.

### Ecosystem services potential: normalization and spatial–temporal assessments

To establish an equivalency between services, a normalization was used to harmonize biophysical values into a common comparable scale. The normalized ES potential raster was calculated for 1990, 2000, 2006, 2012 and 2018, ranging from 0 (no potential supply) to 1 (maximum potential supply). This procedure allowed us to quantify the temporal changes to ES obtained from spatial modelling. Following that, spatial variations in the study area between 1990 and 2018 considering the normalized ES potential (CES) were obtained (Eq. 1):

1 $${\text{CESx}} = \frac{{{\text{ES}}2018{\text{x}} - {\text{ES}}1990{\text{x}}}}{{{\text{ES}}1990{\text{x}}}}*100$$

where CES_x_ is the ES change index for delivering ES of type *x*, ES_1990x_ is the baseline situation for delivering ES of type *x* in 1990, and ES_2018x_ is the situation for delivering ES of type *x* in 2018. To represent spatial shifts, little or no changes in ES correspond to variations between − 0.5 to 0.5 standard deviations between these years. A decline in ES corresponds to variations between − 1.5 and − 0.5 standard deviations (in orange) and higher than − 1.5 standard deviations (in red). A positive change to ES corresponds to variations from 0.5 to 1.5 standard deviations (in light green) and to more than 1.5 standard deviations (in dark green). A one-way ANOVA test was performed for the mean differences among the five observed years (1990, 2000, 2006, 2012, 2018), according to the normality assumptions of Shapiro–Wilk tests, using R^76^.

### The ASEBIO index: an integrated spatial modelling ecosystem services index considering stakeholders’ perceptions

We proposed an integrated index to monitor ES in mainland Portugal based on land cover information and stakeholders’ knowledge. The individual calculation for the ES indicators described above were combined into an ES model potential map with 100 m resolution using a map algebra procedure (Eq. 2):

2 $$ASEBIO_{Index} = \mathop \sum \limits_{i = 1}^{n} ES*w_{i}$$

where *i* is an ES indicator, *w* is the weight of relative importance given by stakeholders for the target ES, and *n* is the number of ES indicators.

The weights of the relative importance of ES embedded in the ASEBIO index equation were taken from previous stakeholders’ workshop sessions led by our team, detailed in^37^. The overarching goal of this meeting was to assess expert perceptions on the ES availability in Portugal, in relation to their background and main interest in this topic. The session had a total of 30 participants, from regional and central administration (12), academia (9), industry (4), politics (3), and non-governmental organizations (2). These stakeholders are experts in various ES categories, not limited to a single area of specialization. To obtain those weights, we implemented a method for ranking ES: values from a 9-step-scale from 0 (not important at all) to 9 (extremely important) were scored by the stakeholders and ES were ranked using an Analytical Hierarchy Processing (AHP)^77^, which is a classification methodology used in multi-criteria analysis, to represent the relevance perceived by stakeholders regarding each one of the eight ES assessed in this study. According to the results, the most relevant ES is drought regulation (0.17) and, conversely, recreation is associated with the least important service (0.04). Hence, the harmonized ES indicators outputs from the spatial modelling were incorporated into the ASEBIO index with weights reflecting the importance of each ES, in view of the stakeholders’ perceptions (Table S9, supplementary information).

Finally, the ASEBIO index was applied following the changes to the ES potential indicators between 1990 and 2018, and a one-way ANOVA test was used to show the variation among the mean values obtained for each year. Shapiro–Wilk tests were also used to determine the normality of data, using R software^76^.

### Comparison of ASEBIO index and ecosystem services potential perceived by stakeholders

From a second exercise in the previously described workshop session, we took the stakeholders’ perceptions about ES potential: participants scored the potential of each land cover class to deliver each ES on a 5-point scale from 0 (no potential at all) to 5 (maximum potential). We provided stakeholders with a reference booklet containing comprehensive descriptions, images, and examples of each CORINE Land Cover class to improve the accuracy of their assessments. Average values determined by the geometric mean were aggregated and the normalization method was applied to each of the 8 ES assessed. A consistency ratio was computed to evaluate the reliability of the stakeholders’ judgments. The scoring process was designed to maintain all assessments on a common scale, facilitating further comparison between both methods. To obtain a combined stakeholders’ ES perception map, we used the same equation with reference to the ASEBIO index.

To compare ES quantified by spatial modelling and stakeholders’ ES potential indicators and overall indices, we calculated the differences between the two approaches for the year 2018, which was feasible due to the application of the common CORINE data. The Pairwise Pearson’s correlation coefficient was calculated for the pair of rasters, using the “ggcorr” function of the “GGally” extension from the R package “ggplot2”^78^. In addition, a paired *t*-test was used to show the statistical difference between the means obtained by the ES models and the stakeholders’ perceptions. Statistical analyses were performed through the R software^76^. The comparison between spatial models’ and stakeholders’ ES potential for 2018 was determined by the average value found in ES models for mainland Portugal.

## Data Availability

The datasets generated during the current study are available from the corresponding author on reasonable request. Output rasters are available in the open repository Figshare and can be downloaded at 10.6084/m9.figshare.26172763.v2.

## jakjd

Supplementary Information.

## References

[1] Costanza, R. et al. Changes in the global value of ecosystem services. *Glob. Environ. Chang.* **26**, 152–158 (2014).

[2] Díaz, S. et al. Pervasive human-driven decline of life on Earth points to the need for transformative change. *Science* **366**, eaax3100 (2019). 31831642 10.1126/science.aax3100

[3] Wood, S. L. R. et al. Distilling the role of ecosystem services in the sustainable development goals. *Ecosyst. Serv.* **29**, 70–82 (2018).

[4] Daw, T., Brown, K., Rosendo, S. & Pomeroy, R. Applying the ecosystem services concept to poverty alleviation: The need to disaggregate human well-being. *Environ. Conserv.* **38**, 370–379 (2011).

[5] Chicago, L. Q., Echeverría, C. & Pizarro, C. J. Ecosystem services trade-offs in landscapes: Trends, areas of greatest impact, and temporal evolution of the scientific field. *Landsc. Ecol.* **37**, 2225–2239 (2022).

[6] Lavorel, S. et al. Pathways to bridge the biophysical realism gap in ecosystem services mapping approaches. *Ecol. Indic.* **74**, 241–260 (2017).

[7] Abe, H., Mitsui, S. & Yamano, H. Conservation of the coral community and local stakeholders’ perceptions of climate change impacts: Examples and gap analysis in three Japanese national parks. *Ocean Coast Manag.* **218**, 106042 (2022).

[8] Aryal, K., Maraseni, T. & Apan, A. How much do we know about trade-offs in ecosystem services? A systematic review of empirical research observations. *Sci. Total Environ.* **806**, 151229 (2022). 34715235 10.1016/j.scitotenv.2021.151229

[9] Koschke, L., Fürst, C., Frank, S. & Makeschin, F. A multi-criteria approach for an integrated land-cover-based assessment of ecosystem services provision to support landscape planning. *Ecol. Indic.* **21**, 54–66 (2012).

[10] Newbold, T. et al. Global effects of land use on local terrestrial biodiversity. *Nature* **520**, 45–50 (2015). 25832402 10.1038/nature14324

[11] Dabalà, A. et al. Priority areas to protect mangroves and maximise ecosystem services. *Nat. Commun.* **14**, 5863 (2023). 37735160 10.1038/s41467-023-41333-3 PMC10514197

[12] Sagie, H. & Orenstein, D. E. Benefits of Stakeholder integration in an ecosystem services assessment of Mount Carmel biosphere reserve, Israel. *Ecosyst. Serv.* **53**, 101404 (2022).

[13] Vallet, A. et al. Relationships between ecosystem services: Comparing methods for assessing tradeoffs and synergies. *Ecol. Econ.* **150**, 96–106 (2018).

[14] Harrison, P. A. et al. Selecting methods for ecosystem service assessment: A decision tree approach. *Ecosyst. Serv.* **29**, 481–498 (2018).

[15] Braun, D., Damm, A., Hein, L., Petchey, O. L. & Schaepman, M. E. Spatio-temporal trends and trade-offs in ecosystem services: An earth observation based assessment for Switzerland between 2004 and 2014. *Ecol. Indic.* **89**, 828–839 (2018).

[16] Rukundo, E. et al. Spatio-temporal dynamics of critical ecosystem services in response to agricultural expansion in Rwanda, East Africa. *Ecol. Indic.* **89**, 696–705 (2018).

[17] Renard, D., Rhemtulla, J. M., Bennett, E. M., Rhemtull, J. M. & Bennett, E. M. Historical dynamics in ecosystem service bundles. *Proc. Natl. Acad. Sci.* **112**, 13411–13416 (2015). 26460005 10.1073/pnas.1502565112 PMC4629320

[18] Musche, M. et al. Research questions to facilitate the future development of European long-term ecosystem research infrastructures: A horizon scanning exercise. *J. Environ. Manage.* **250**, 109479 (2019). 31499467 10.1016/j.jenvman.2019.109479

[19] Maes, J. et al. Mapping ecosystem services for policy support and decision making in the European Union. *Ecosyst. Serv.* **1**, 31–39 (2012).

[20] Bagstad, K. J., Semmens, D. J., Waage, S. & Winthrop, R. A comparative assessment of decision-support tools for ecosystem services quantification and valuation. *Ecosyst. Serv.* **5**, 27–39 (2013).

[21] Grêt-Regamey, A., Sirén, E., Brunner, S. H. & Weibel, B. Review of decision support tools to operationalize the ecosystem services concept. *Ecosyst. Serv.* **26**, 306–315 (2017).

[22] Haase, D., Schwarz, N., Strohbach, M., Kroll, F. & Seppelt, R. Synergies, trade-offs, and losses of ecosystem services in urban regions: An integrated multiscale framework applied to the Leipzig-Halle region, Germany. *Ecol. Soc.* **17**, 22 (2012).

[23] Sharp, R. et al. *InVEST 3.10.2 User’s Guide.* The Natural Capital Project, Stanford University, University of Minnesota, The Nature Conservancy, World Wildlife Fund (2020).

[24] Sánchez-Canales, M. et al. Sensitivity analysis of ecosystem service valuation in a Mediterranean watershed. *Sci. Total Environ.* **440**, 140–153 (2012). 22925484 10.1016/j.scitotenv.2012.07.071

[25] Redhead, J. W. et al. Empirical validation of the InVEST water yield ecosystem service model at a national scale. *Sci. Total Environ.* **569–570**, 1418–1426 (2016). 27395076 10.1016/j.scitotenv.2016.06.227

[26] Graça, M. et al. Assessing how green space types affect ecosystem services delivery in Porto, Portugal. *Landsc. Urban Plan* **170**, 195–208 (2018).

[27] Elliot, T., Almenar, J. B. & Rugani, B. Impacts of policy on urban energy metabolism at tackling climate change: The case of Lisbon. *J. Clean. Prod.* **276**, 123510 (2020).

[28] Vaz, A. S. et al. Integrating conservation targets and ecosystem services in landscape spatial planning from Portugal. *Landsc. Urban Plan.* **215**, 104213 (2021).

[29] Mascarenhas, A., Haase, D., Ramos, T. B. & Santos, R. Pathways of demographic and urban development and their effects on land take and ecosystem services: The case of Lisbon metropolitan area, Portugal. *Land Use Policy* **82**, 181–194 (2019).

[30] Carvalho-Santos, C. et al. Ecosystem services in a protected mountain range of Portugal: Satellite-based products for state and trend analysis. *Remote Sens. (Basel)* **10**, 1573 (2018).

[31] Clemente, P. et al. Combining social media photographs and species distribution models to map cultural ecosystem services: The case of a Natural Park in Portugal. *Ecol. Indic.* **96**, 59–68 (2019).

[32] Mascarenhas, A., Ramos, T. B., Haase, D. & Santos, R. Participatory selection of ecosystem services for spatial planning: Insights from the Lisbon metropolitan area, Portugal. *Ecosyst. Serv.* **18**, 87–99 (2016).

[33] Terêncio, D. P. S. et al. Integrating ecosystem services into sustainable landscape management: A collaborative approach. *Sci. Total Environ.* **794**, 148538 (2021). 34323777 10.1016/j.scitotenv.2021.148538

[34] Dechazal, J., Quetier, F., Lavorel, S. & Vandoorn, A. Including multiple differing stakeholder values into vulnerability assessments of socio-ecological systems. *Global Environ. Change* **18**, 508–520 (2008).

[35] Wentling, C., Campos, F. S., David, J. & Cabral, P. Pollination potential in Portugal: Leveraging an ecosystem service for sustainable agricultural productivity. *Land (Basel)* **10**, 431 (2021).

[36] Campos, F. S. et al. The economic and ecological benefits of saving ecosystems to protect services. *J. Clean. Prod.* **311**, 127551 (2021).

[37] Cabral, P., Campos, F. S., David, J. & Caser, U. Disentangling ecosystem services perception by stakeholders: An integrative assessment based on land cover. *Ecol. Indic.* **126**, 107660 (2021).

[38] Campagne, C. S., Roche, P., Müller, F. & Burkhard, B. T. Ten years of ecosystem services matrix: Review of a (r)evolution. *One Ecosyst.* **5**, e51103 (2020).

[39] Parente, J., Tonini, M., Amraoui, M. & Pareira, M. Socioeconomic impacts and regional drivers of fire management: The case of Portugal. In *Fire Hazards: Socio-Economic and Regional Issues* 181–194 (Springer International Publishing, 2024). 10.1007/978-3-031-50446-4_14.

[40] Pacheco, R. M. Carbon taxation as a means to incentivize forest and fire management. *Environ. Dev. Sustain.* **24**, 12387–12403 (2022).

[41] Campos, J. C. et al. Climate regulation ecosystem services and biodiversity conservation are enhanced differently by climate- and fire-smart landscape management. *Environ. Res. Lett.* **17**, 054014 (2022).

[42] Schägner, J. P. et al. Spatial dimensions of recreational ecosystem service values: A review of meta-analyses and a combination of meta-analytic value-transfer and GIS. *Ecosyst. Serv.* **31**, 395–409 (2018).

[43] Parente, J., Amraoui, M., Menezes, I. & Pereira, M. G. Drought in Portugal: Current regime, comparison of indices and impacts on extreme wildfires. *Sci. Total Environ.* **685**, 150–173 (2019). 31174114 10.1016/j.scitotenv.2019.05.298

[44] Chaplin-Kramer, R. et al. Global modeling of nature’s contributions to people. *Science* **1979**(366), 255–258 (2019). 10.1126/science.aaw3372 31601772

[45] Palmero-Iniesta, M., Espelta, J. M., Gordillo, J. & Pino, J. Changes in forest landscape patterns resulting from recent afforestation in Europe (1990–2012): Defragmentation of pre-existing forest versus new patch proliferation. *Ann. For. Sci.* **77**, 43 (2020).

[46] Plieninger, T., Torralba, M., Hartel, T. & Fagerholm, N. Perceived ecosystem services synergies, trade-offs, and bundles in European high nature value farming landscapes. *Landsc. Ecol.* **34**, 1565–1581 (2019).

[47] Oliveira, T. M., Guiomar, N., Baptista, F. O., Pereira, J. M. C. & Claro, J. Is Portugal’s forest transition going up in smoke?. *Land Use Policy* **66**, 214–226 (2017).

[48] Copernicus. CORINE Land cover. https://land.copernicus.eu/ (2018).

[49] Petersen, J. E., Mancosu, E. & King, S. Ecosystem extent accounts for Europe. *Ecosyst. Serv.* **57**, 101457 (2022).

[50] Zheng, H. et al. Distinguishing the impacts of land use change in intensity and type on ecosystem services trade-offs. *J. Environ. Manage.* **316**, 115206 (2022). 35597216 10.1016/j.jenvman.2022.115206

[51] Evans, D. L. et al. Ecosystem service delivery by urban agriculture and green infrastructure—a systematic review. *Ecosyst. Serv.* **54**, 101405 (2022).

[52] Nicolau, R., David, J., Caetano, M. & Pereira, J. Ratio of land consumption rate to population growth rate—analysis of different formulations applied to Mainland Portugal. *ISPRS Int. J. Geoinf.* **8**, 10 (2018).

[53] Grimm, N. B. et al. Global change and the ecology of cities. *Science* **1979**(319), 756–760 (2008). 10.1126/science.1150195 18258902

[54] Jenkins, M. Prospects for biodiversity. *Science* **1979**(302), 1175–1177 (2003). 10.1126/science.1088666 14615529

[55] Gómez-Baggethun, E. & Barton, D. N. Classifying and valuing ecosystem services for urban planning. *Ecol. Econ.* **86**, 235–245 (2013).

[56] Schirpke, U. & Tasser, E. Trends in ecosystem services across Europe due to land-use/cover changes. *Sustainability* **13**, 7095 (2021).

[57] Zoderer, B. M., Tasser, E., Carver, S. & Tappeiner, U. Stakeholder perspectives on ecosystem service supply and ecosystem service demand bundles. *Ecosyst. Serv.* **37**, 100938 (2019).

[58] Posner, S., Verutes, G., Koh, I., Denu, D. & Ricketts, T. Global use of ecosystem service models. *Ecosyst. Serv.* **17**, 131–141 (2016).

[59] Pelorosso, R., Apollonio, C., Rocchini, D. & Petroselli, A. Effects of land use-land cover thematic resolution on environmental evaluations. *Remote Sens. (Basel)* **13**, 1232 (2021).

[60] Hein, L., van Koppen, K., de Groot, R. S. & van Ierland, E. C. Spatial scales, stakeholders and the valuation of ecosystem services. *Ecol. Econ.* **57**, 209–228 (2006).

[61] Stosch, K. C., Quilliam, R. S., Bunnefeld, N. & Oliver, D. M. Quantifying stakeholder understanding of an ecosystem service trade-off. *Sci. Total Environ.* **651**, 2524–2534 (2019). 30340188 10.1016/j.scitotenv.2018.10.090

[62] Elliott, R. M. et al. Identifying linkages between urban green infrastructure and ecosystem services using an expert opinion methodology. *Ambio* **49**, 569–583 (2020). 31473977 10.1007/s13280-019-01223-9 PMC6965533

[63] Xu, Z. & Peng, J. Ecosystem services-based decision-making: A bridge from science to practice. *Environ. Sci. Policy* **135**, 6–15 (2022).

[64] Chaplin-Kramer, R. et al. Conservation needs to integrate knowledge across scales. *Nat. Ecol. Evol.* **6**, 118–119 (2022). 34824390 10.1038/s41559-021-01605-x

[65] Ramirez-Reyes, C. et al. Reimagining the potential of earth observations for ecosystem service assessments. *Sci. Total Environ.* **665**, 1053–1063 (2019). 30893737 10.1016/j.scitotenv.2019.02.150

[66] Scowen, M., Athanasiadis, I. N., Bullock, J. M., Eigenbrod, F. & Willcock, S. The current and future uses of machine learning in ecosystem service research. *Sci. Total Environ.* **799**, 149263 (2021). 34426354 10.1016/j.scitotenv.2021.149263

[67] INE. Census provisional results. https://www.ine.pt/ngt_server/attachfileu.jsp?look_parentBoui=539114868&att_display=n&att_download=y (2021).

[68] Portela, M. M., Espinosa, L. A. & Zelenakova, M. Long-term rainfall trends and their variability in Mainland Portugal in the last 106 years. *Climate* **8**, 146 (2020).

[69] Belo-Pereira, M., Dutra, E. & Viterbo, P. Evaluation of global precipitation data sets over the Iberian Peninsula. *J. Geophys. Res. Atmos.* **116**, 1–16 (2011).

[70] Mingarro, M. & Lobo, J. M. European National Parks protect their surroundings but not everywhere: A study using land use/land cover dynamics derived from CORINE land cover data. *Land Use Policy* **124**, 106434 (2023).

[71] Paprotny, D., Terefenko, P., Giza, A., Czapliński, P. & Vousdoukas, M. I. Future losses of ecosystem services due to coastal erosion in Europe. *Sci. Total Environ.* **760**, 144310 (2021). 33341636 10.1016/j.scitotenv.2020.144310

[72] Maes, J. et al. Accounting for forest condition in Europe based on an international statistical standard. *Nat. Commun.* **14**, 3723 (2023). 37349309 10.1038/s41467-023-39434-0 PMC10287664

[73] Spyra, M., Kleemann, J., Calò, N. C., Schürmann, A. & Fürst, C. Protection of peri-urban open spaces at the level of regional policy-making: Examples from six European regions. *Land Use Policy* **107**, 105480 (2021).

[74] Sousa, L. P., Sousa, A. I., Alves, F. L. & Lillebø, A. I. Ecosystem services provided by a complex coastal region: Challenges of classification and mapping. *Sci. Rep.* **6**, 22782 (2016). 26964892 10.1038/srep22782 PMC4786800

[75] Eurostat. NUTS. https://ec.europa.eu/eurostat/web/gisco/geodata/reference-data/administrative-units-statistical-units/nuts.

[76] R Development Core Team. *R Foundation for Statistical Computing*. http://www.r-project.org/.

[77] Saaty, R. W. The analytic hierarchy process-what it is and how it is used. *Math. Modell.* **9**, 161–176 (1987).

[78] Schloerke, B. et al. Ggally: Extension to ggplot2. *R package version 2.1* (2021).

