# Supplementary material for: Humans versus models: a comparative assessment of ecosystem services models and stakeholders’ perceptions
Source: Sci Rep. 2024 Oct 29;14:25995. doi: 10.1038/s41598-024-76600-w (PMC11522275; doi:10.1038/s41598-024-76600-w)
Supplement: Supplementary file 1 — Supplementary Information. [file 41598_2024_76600_MOESM1_ESM.docx]

**Supplementary Information**

**Humans vs models: a comparative assessment of ecosystem services models and stakeholders**’ **perceptions**

João David^1*^, Pedro Cabral^2*,3^, Felipe S. Campos ^4,5,3^

^1^Humboldt-Universität zu Berlin, Geography Department, Landscape Ecology Lab, Rudower Chaussee 16, 12489, Berlin, Germany

^2^School of Remote Sensing and Geomatics Engineering, Nanjing University of Information Science and Technology, Nanjing 210044, China

^3^NOVA Information Management School (NOVA IMS), Universidade Nova de Lisboa, Campus de Campolide, 1070-312 Lisboa, Portugal

^4^Universitat Autònoma de Barcelona, 08193 Cerdanyola del Vallès, Catalunya, Spain

^5^Centre de Recerca Ecològica i Aplicacions Forestals (CREAF), 08193 Cerdanyola del Vallès, Catalunya, Spain

Correspondence: J.D. (joao.david@geo.hu-berlin.de) or P.C. (cabral@nuist.edu.cn)

**Supplementary Figure**


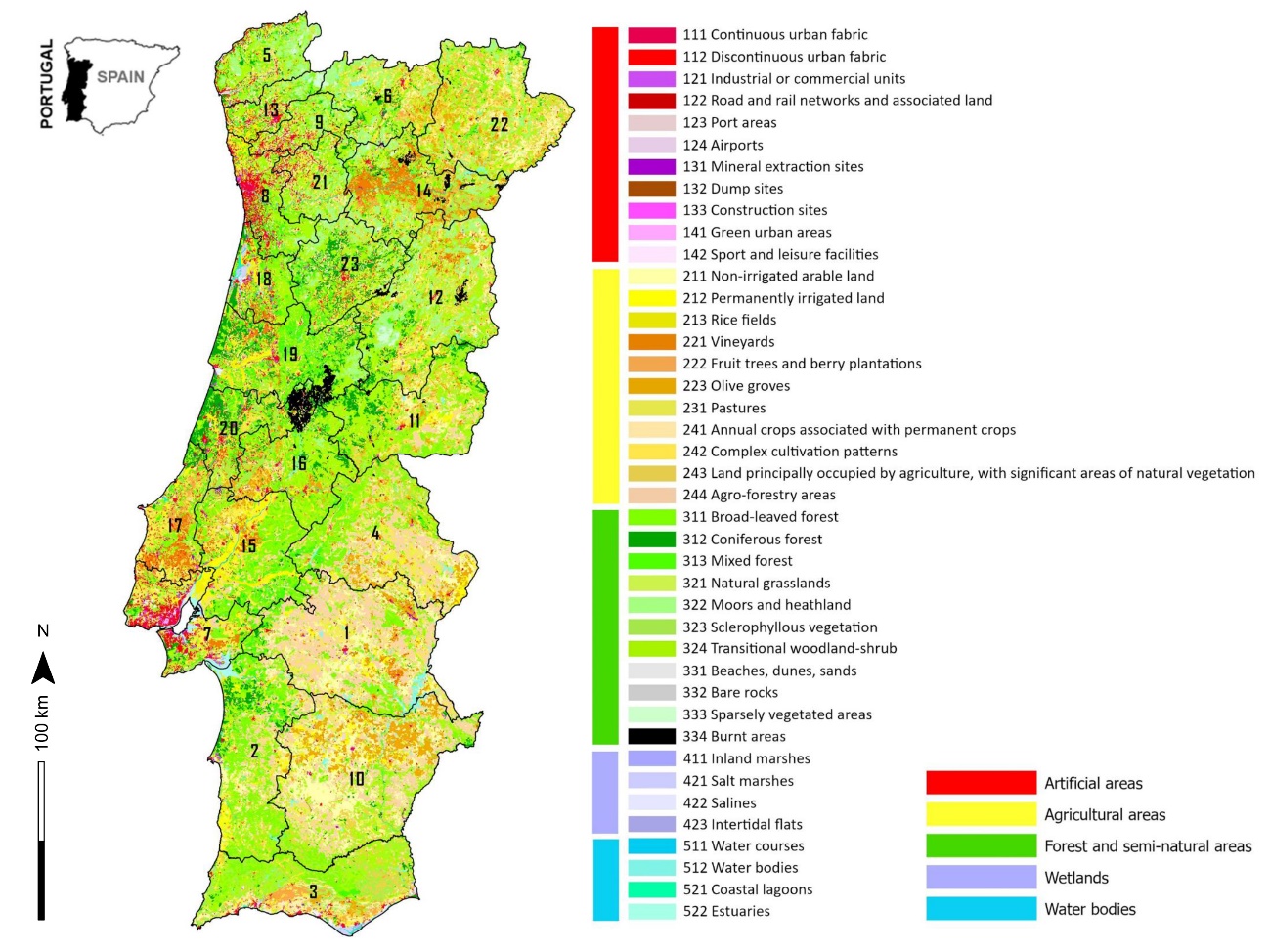


**Fig. S1.**Study area with land cover classes for 2018. Data source: CORINE Land Cover ^1^. NUTS 3 regions: 1. Alentejo Central, 2. Alentejo Litoral, 3. Algarve; 4. Alto Alentejo, 5. Alto Minho, 6. Alto Tâmega, 7. Área Metropolitana de Lisboa, 8. Área Metropolitana de Porto, 9. Ave, 10. Baixo Alentejo, 11. Beira Baixa, 12. Beiras e Serra da Estrela, 13. Cávado, 14. Douro, 15. Lezíria do Tejo, 16. Médio Tejo, 17. Oeste, 18. Região de Aveiro, 19. Região de Coimbra, 20. Região de Leiria, 21. Tâmega e Sousa, 22. Terras de Trás-os-Montes, 23. Viseu Dão Lafões).

**Supplementary Text**

**Fig.4 Legend**

**
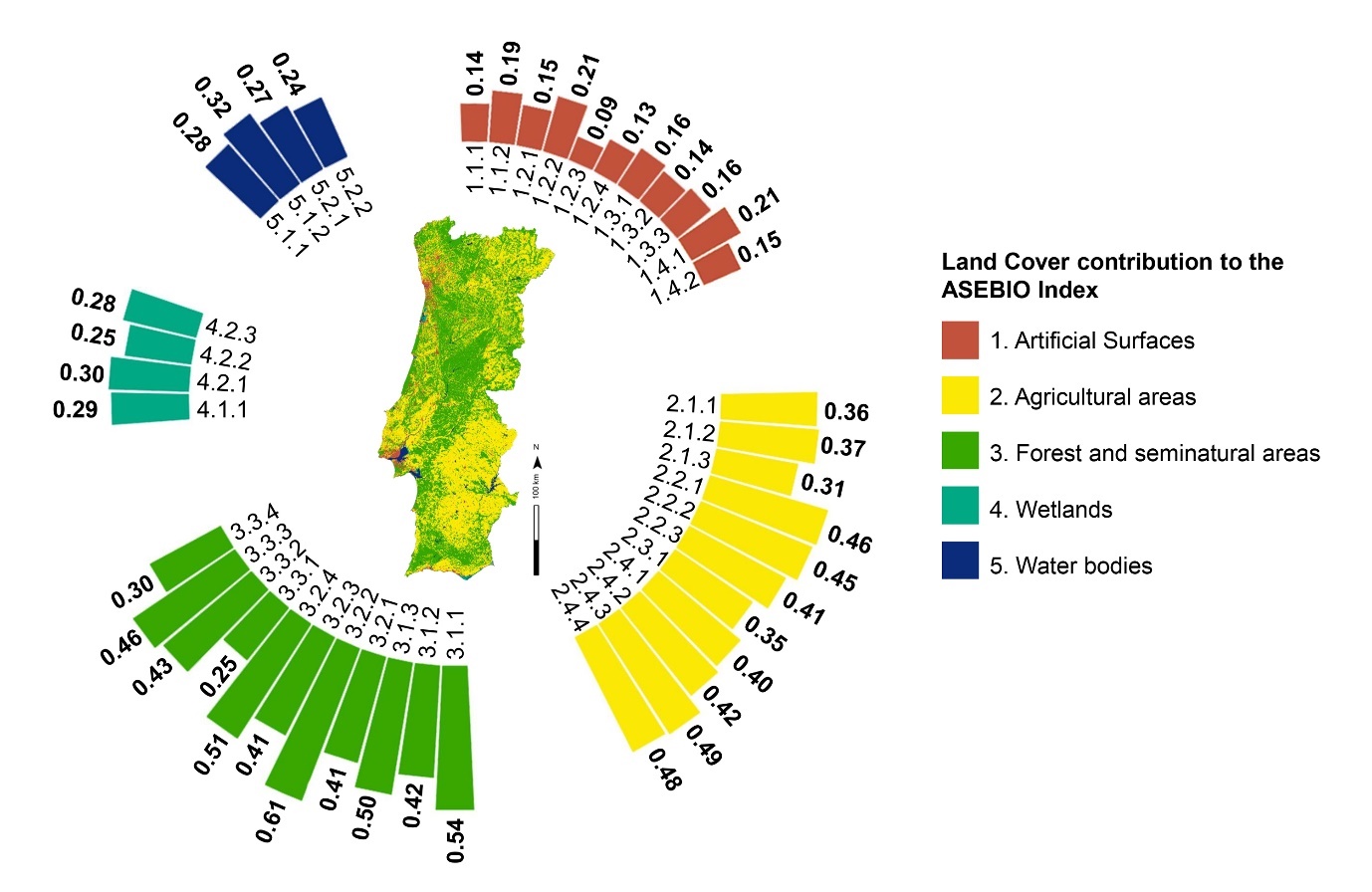
**

**Fig. 4.**Land cover contribution to the ASEBIO index in 2018. Average values by land cover classes are represented according to the CORINE level 3 nomenclature: 1.1.1. Continuous urban fabric; 1.1.2. Discontinuous urban fabric 1.2.1. Industrial or commercial units; 1.2.2. Road and rail networks and associated land; 1.2.3. Port areas; 1.2.4. Airports; 1.3.1. Mineral extraction sites; 1.3.2. Dump sites; 1.3.3. Construction sites; 1.4.1. Green urban areas; 1.4.2. Sport and leisure facilities; 2.1.1. Non-irrigated arable land; 2.1.2. Permanently irrigated land; 2.1.3. Rice fields; 2.2.1. Vineyards; 2.2.2. Fruit trees and berry plantations; 2.2.3. Olive groves; 2.3.1. Pastures; 2.4.1. Annual crops associated with permanent crops; 2.4.2. Complex cultivation patterns; 2.4.3. Land principally occupied by agriculture, with significant areas of natural vegetation; 2.4.4. Agro-forestry areas; 3.1.1. Broad-leaved forest; 3.1.2. Coniferous forest; 3.1.3. Mixed forest; 3.2.1. Natural grasslands; 3.2.2. Moors and heathland; 3.2.3. Sclerophyllous vegetation; 3.2.4. Transitional woodland-shrub; 3.3.1. Beaches, dunes, sands; 3.3.2. Bare rocks; 3.3.3. Sparsely vegetated areas; 3.3.4. Burnt areas; 4.1.1. Inland marshes; 4.2.1. Salt marshes; 4.2.2. Salines; 4.2.3. Intertidal flats; 5.1.1. Water courses; 5.1.2. Water bodies; 5.2.1. Coastal lagoons; 5.2.2. Estuaries

**Rationale for Calculating the Selected Ecosystem Services Indicators**

**Climate Regulation**

Carbon sequestration is a valuable global climate regulation service ^2^. We used the InVEST Carbon model ^3^to quantify the amount of carbon stored by each land use land cover class in four carbon pools: aboveground biomass and belowground biomass, soil, and dead organic matter. Estimates for each of the carbon pools were obtained from averaging values found in the literature for Portugal ^4–6^. When these estimations were unavailable, we collected values from studies in Spain, using the same calculations ^7–10^. Only a few carbon soil values were still missing for some land cover classes, and these were assigned from a report by the Intergovernmental Panel on Climate Change ^11^. The estimated carbon stored in the Portuguese landscape is reported in t/ha/year. Carbon sequestration between 1990 and 2018 was measured.

**Water Purification**

Nutrient retention assesses the LULC effects on water quality, providing information about which areas most effectively purify water sources for people ^3^. The nutrient retention data was calculated using the InVEST nutrient delivery model, which is used to map nitrogen from watersheds and its transport into the streams based on land cover ^3,12^. Datasets used in this model include a digital elevation model with a spatial resolution of 25m ^13^, annual average precipitation rasters functioning as a nutrient runoff proxy weighting factor ^14^, watersheds polygons ^15^, and biophysical parameters for Portugal. For the latter variable, the average annual quantity of nutrients exported from each LULC cell was determined using values found in the literature for nitrogen coefficients, providing good coverage of all major land cover categories in Portugal ^3,16–25^. The nutrient load was obtained by routing water along flow paths based on slope ^21^. After that, the nutrient load quantity retained by the landscape was determined using the nutrient retention capacity of each LULC ^3^. Therefore, nutrient output retention maps reflect the mitigated pollution and were estimated by subtracting nitrogen load and nutrient export values ^26^.

**Habitat Quality**

We used the InVEST Habitat Quality (HQ) model ^3^ as an ecological proxy for the conservation status of the ecosystems. Model outputs represent the potential of biodiversity responses to human-induced landscape changes. Thus, pixel values range from 0 to 1, where 0 is not suitable and 1 expresses maximum habitat suitability. In our case, the suitability values are affected by three main factors: 1.) the relative sensitivity of each land cover class in terms of threats to habitats (i.e., buildings, artificial pastures, intensive farming, railways, and roads); 2.) the relative impact of each threat to the ecosystems (i.e., habitat responses); and 3.) the relative distance between ecosystems and threats across the landscape, according to a distance-decay rate over the spatial scale under investigation ^3^.

**Drought Regulation**

Water yield is the amount of water running off the landscape ^3^. The Annual Water Yield InVEST model is based on the traditional Budyko curve (ω = 2.6) and annual average precipitation rates ^3^. To calculate the average annual water yield, we used a root restriction layer depth and a plant available water content raster from the European Soil Data Centre ^27^, a reference evapotranspiration raster from the Consortium of Spatial Information ^28^, an annual precipitation raster from the WorldClim database ^14^, and watersheds polygons produced by the National Information System of Hydric Resources ^15^. The root depths and evapotranspiration biophysical coefficients were obtained from Almeida & Cabral ^29^. Water yield outputs are reported in m^3^/ha/year.

**Recreation**

The recreation model identifies high-quality areas for nature recreation. This ES indicator was calculated using an adapted version of Vallecillo *et al.* ^30^ and Zulian *et al.* ^31^ where the authors applied a spatially explicit framework to create a recreation potential index using defined factors. Our modelling process considers a land cover suitability score based on expert knowledge ^30^, the presence/absence of national protected areas ^32^, the distance from residential areas ^1^, and the distance to the coast and sea ^1^. We computed a weighted sum technique in the ArcGIS Pro 3.2 ^33^ scaled from 0-1 to overlay all datasets into an integrated result: suitability score weighted 0.6, protected areas 0.2, and the remaining two layers 0.1. Output results show the recreation potential from high to low at the pixel level (100m). Areas with greater capacity for the recreation service have a higher potential ^30^.

**Food Provisioning**

The total area of each land cover class dedicated to agriculture is summed and used as a proxy of the food provisioning service, as suggested by Cabral *et al.* ^34^. In this simplified approach, the Food Provisioning model estimates the agricultural area for a fixed set of crops, derived from the land cover information. Then, the observed values as outputs are reported as the total land (ha) dedicated to agriculture. We included arable land, permanent crops, pastures, and heterogeneous agricultural classes from the CORINE land cover maps ^1^ .

**Erosion Prevention**

Soil erosion is a natural process and can be caused by natural processes such as rain and irrigation. The Sediment Retention InVEST model ^3^ was used to determine the ability of the landscape to retain sediments in watersheds ^15^ as a function rainfall erosivity ^35^, soil erodibility ^36^, slope length ^13^, cover management factor ^37^, and supporting practice factor ^37^. The cover management factor expresses the cropping effects on erosion rates ^38^ and the supporting practice factor settles for control practices which reduces the runoff erosion potential ^39^. The model uses the Revised Universal Soil Loss Equation (RUSLE) to calculate the amount of soil loss on each LULC ^3^. Outputs show the mitigated erosion, reported in t/ha/year.

**Pollination**

The InVEST crop pollination model produces pollinator supply indices ranging from 0 (least suitable) to 1 (most suitable), describing the likelihood of pollinators using a space, based on proximal nesting sites, food resources and foraging ranges of the species ^3^. The results characterize the consistency of land to host European honeybees (*Apis mellifera*), which are economically valuable pollinators. The model uses biological guild tables as inputs ^1,40^, incorporating habitat parameters, estimating nesting sites and vegetation availability, and relative abundance per guild for each cell of the input raster, considering the vegetation parameters of its neighbours ^1,40^. The model also considers foraging distances to bridge the possible spatial separations of nesting and foraging habitats ^41–43^.

**Supplementary Tables**

**Ecosystem Services Indicators - Data Parameters**

**Table S1 – Climate Regulation**

| **CARBON POOLS** | | | | |
| --- | --- | --- | --- | --- |
| **Land Cover Class** | **Carbon Above Biomass** | **Carbon Below Biomass** | **Carbon Soil** | **Dead Organic Matter** |
| Continuos urban fabric | 0.00 | 0.00 | 0.00 | 0.00 |
| Discontinuous urban fabric | 0.00 | 0.00 | 3.23 | 0.00 |
| Industrial or commercial units | 0.00 | 0.00 | 0.00 | 0.00 |
| Road and rail networks and associated land | 0.00 | 0.00 | 0.00 | 0.00 |
| Port areas | 0.00 | 0.00 | 0.00 | 0.00 |
| Airports | 0.00 | 0.00 | 0.50 | 0.00 |
| Mineral extraction sites | 0.00 | 0.00 | 0.00 | 0.00 |
| Dump Sites | 0.00 | 0.00 | 0.00 | 0.00 |
| Construction Sites | 0.00 | 0.00 | 0.00 | 0.00 |
| Green urban areas | 0.00 | 0.00 | 6.46 | 0.00 |
| Sport and Leisure facilities | 0.00 | 0.00 | 9.42 | 0.00 |
| Non-irrigated arable land | 0.31 | 0.31 | 38.00 | 0.00 |
| Permanently irrigated land | 0.31 | 0.31 | 54.00 | 0.00 |
| Rice Fields | 0.00 | 0.00 | 5.00 | 0.00 |
| Vineyards | 2.84 | 2.22 | 35.50 | 0.00 |
| Fruit trees and berry plantations | 0.80 | 0.40 | 31.00 | 0.00 |
| Olive groves | 7.85 | 1.15 | 43.00 | 0.00 |
| Pastures | 8.46 | 1.48 | 6.00 | 0.00 |
| Annual crops associated with permanent crops | 0.00 | 0.00 | 13.00 | 0.00 |
| Complex cultivation patterns | 2.09 | 1.05 | 31.00 | 0.00 |
| Land principally occupied by agriculture, with significant areas of natural vegetation | 0.00 | 0.00 | 11.37 | 0.00 |
| Agro-forestry areas | 22.91 | 5.54 | 61.53 | 1.44 |
| Broad-leaved forest | 36.67 | 21.10 | 120.00 | 1.88 |
| Coniferous forest | 18.22 | 2.85 | 73.66 | 3.26 |
| Mixed Forest | 39.95 | 21.56 | 120.00 | 2.43 |
| Natural grasslands | 0.00 | 0.00 | 3.40 | 0.00 |
| Moors and heathland | 9.07 | 5.15 | 106.64 | 1.57 |
| Sclerophyllous vegetation | 0.00 | 0.00 | 17.74 | 0.00 |
| Transitional woodland-shrub | 8.47 | 4.46 | 109.57 | 0.72 |
| Beaches, dunes, sands | 0.00 | 0.00 | 0.00 | 0.00 |
| Bare rocks | 1.63 | 3.05 | 34.50 | 0.35 |
| Sparsely vegetated areas | 0.00 | 0.00 | 1.52 | 0.00 |
| Burnt areas | 0.00 | 0.00 | 1.52 | 0.00 |
| Inland marshes | 0.00 | 0.00 | 1.50 | 0.00 |
| Salt Marshes | 0.00 | 0.00 | 2.00 | 0.00 |
| Salines | 0.00 | 0.00 | 0.00 | 0.00 |
| Intertidal flats | 0.00 | 0.00 | 0.00 | 0.00 |
| Water courses | 0.00 | 0.00 | 0.00 | 0.00 |
| Water bodies | 0.00 | 0.00 | 0.00 | 0.00 |
| Coastal lagoons | 0.00 | 0.00 | 0.00 | 0.00 |
| Estuaries | 0.00 | 0.00 | 0.00 | 0.00 |

**Table S2 – Water Purification**

| **BIOPHYSICAL TABLE** | | | | |
| --- | --- | --- | --- | --- |
| **Land Cover Class** | **Nitrogen Load (kg/ha)** | **Maximum Nitrogen Retention Efficiency Ratio** | **Distance to Retain Nitrogen (m)** | **Nitrogen Dissolved into the Subsurface Ratio** |
| Continuos urban fabric | 10 | 0.05 | 10 | 0 |
| Discontinuous urban fabric | 10 | 0.05 | 10 | 0 |
| Industrial or commercial units | 10 | 0.05 | 10 | 0 |
| Road and rail networks and associated land | 10 | 0.05 | 10 | 0 |
| Port areas | 10 | 0.05 | 10 | 0 |
| Airports | 10 | 0.05 | 10 | 0 |
| Mineral extraction sites | 10 | 0.05 | 10 | 0 |
| Dump Sites | 10 | 0.05 | 10 | 0 |
| Construction Sites | 10 | 0.05 | 10 | 0 |
| Green urban areas | 10 | 0.05 | 10 | 0 |
| Sport and Leisure facilities | 10 | 0.05 | 10 | 0 |
| Non-irrigated arable land | 100 | 0.5 | 25 | 0.5 |
| Permanently irrigated land | 100 | 0.5 | 25 | 0.5 |
| Rice Fields | 100 | 0.5 | 25 | 0.5 |
| Vineyards | 100 | 0.5 | 25 | 0.5 |
| Fruit trees and berry plantations | 100 | 0.5 | 25 | 0.5 |
| Olive groves | 100 | 0.5 | 25 | 0.5 |
| Pastures | 8 | 0.75 | 150 | 0 |
| Annual crops associated with permanent crops | 100 | 0.5 | 25 | 0.5 |
| Complex cultivation patterns | 100 | 0.5 | 25 | 0.5 |
| Land principally occupied by agriculture, with significant areas of natural vegetation | 100 | 0.5 | 25 | 0.5 |
| Agro-forestry areas | 100 | 0.5 | 25 | 0.5 |
| Broad-leaved forest | 2.8 | 0.8 | 300 | 0 |
| Coniferous forest | 2.8 | 0.8 | 300 | 0 |
| Mixed Forest | 2.8 | 0.8 | 300 | 0 |
| Natural grasslands | 8 | 0.75 | 150 | 0 |
| Moors and heathland | 8 | 0.75 | 150 | 0 |
| Sclerophyllous vegetation | 8 | 0.75 | 150 | 0 |
| Transitional woodland-shrub | 2.8 | 0.8 | 300 | 0 |
| Beaches, dunes, sands | 8 | 0.75 | 150 | 0 |
| Bare rocks | 8 | 0.75 | 150 | 0 |
| Sparsely vegetated areas | 8 | 0.75 | 150 | 0 |
| Burnt areas | 2.8 | 0.8 | 300 | 0 |
| Inland marshes | 2.8 | 0.8 | 10 | 0 |
| Salt Marshes | 2.8 | 0.8 | 10 | 0 |
| Salines | 2.8 | 0.8 | 10 | 0 |
| Intertidal flats | 2.8 | 0.8 | 10 | 0 |
| Water courses | 2.8 | 0.8 | 10 | 0 |
| Water bodies | 2.8 | 0.8 | 10 | 0 |
| Coastal lagoons | 2.8 | 0.8 | 10 | 0 |
| Estuaries | 2.8 | 0.8 | 10 | 0 |

**Table S3 – Habitat Quality**

**Table S3.1 – Habitat Quality Sensitivity Matrix***

| **Land Cover Class** | **Habitat Suitability** | **Buildings** | **Artificial Pastures** | **Intensive Farming** | **Railways** | **Motorways, Trunks and Primary roads** | **Secondary and tertiary roads** | **Residential and service roads** | **Tracks and bridleways** |
| --- | --- | --- | --- | --- | --- | --- | --- | --- | --- |
| Continuos urban fabric | 0.09 | 0.19 | 0.07 | 0.1 | 0.15 | 0.19 | 0.17 | 0.15 | 0.06 |
| Discontinuous urban fabric | 0.09 | 0.19 | 0.07 | 0.1 | 0.15 | 0.19 | 0.17 | 0.15 | 0.06 |
| Industrial or commercial units | 0.09 | 0.19 | 0.07 | 0.1 | 0.15 | 0.19 | 0.17 | 0.15 | 0.06 |
| Road and rail networks and associated land | 0.09 | 0.19 | 0.07 | 0.1 | 0.15 | 0.19 | 0.17 | 0.15 | 0.06 |
| Port areas | 0.09 | 0.19 | 0.07 | 0.1 | 0.15 | 0.19 | 0.17 | 0.15 | 0.06 |
| Airports | 0.09 | 0.19 | 0.07 | 0.1 | 0.15 | 0.19 | 0.17 | 0.15 | 0.06 |
| Mineral extraction sites | 0.09 | 0.19 | 0.07 | 0.1 | 0.15 | 0.19 | 0.17 | 0.15 | 0.06 |
| Dump Sites | 0.09 | 0.19 | 0.07 | 0.1 | 0.15 | 0.19 | 0.17 | 0.15 | 0.06 |
| Construction Sites | 0.09 | 0.19 | 0.07 | 0.1 | 0.15 | 0.19 | 0.17 | 0.15 | 0.06 |
| Green urban areas | 0.27 | 0.56 | 0.21 | 0.31 | 0.46 | 0.56 | 0.52 | 0.46 | 0.19 |
| Sport and Leisure facilities | 0.27 | 0.56 | 0.21 | 0.31 | 0.46 | 0.56 | 0.52 | 0.46 | 0.19 |
| Non-irrigated arable land | 0.26 | 0.51 | 0.12 | 0 | 0.44 | 0.61 | 0.54 | 0.47 | 0.24 |
| Permanently irrigated land | 0.26 | 0.51 | 0.12 | 0 | 0.44 | 0.61 | 0.54 | 0.47 | 0.24 |
| Rice Fields | 0.26 | 0.51 | 0.12 | 0 | 0.44 | 0.61 | 0.54 | 0.47 | 0.24 |
| Vineyards | 0.26 | 0.51 | 0.12 | 0 | 0.44 | 0.61 | 0.54 | 0.47 | 0.24 |
| Fruit trees and berry plantations | 0.26 | 0.51 | 0.12 | 0 | 0.44 | 0.61 | 0.54 | 0.47 | 0.24 |
| Olive groves | 0.26 | 0.51 | 0.12 | 0 | 0.44 | 0.61 | 0.54 | 0.47 | 0.24 |
| Pastures | 0.26 | 0.51 | 0.12 | 0 | 0.44 | 0.61 | 0.54 | 0.47 | 0.24 |
| Annual crops associated with permanent crops | 0.26 | 0.51 | 0.12 | 0 | 0.44 | 0.61 | 0.54 | 0.47 | 0.24 |
| Complex cultivation patterns | 0.52 | 0.62 | 0 | 0.54 | 0.51 | 0.71 | 0.61 | 0.55 | 0.26 |
| Land principally occupied by agriculture, with significant areas of natural vegetation | 0.52 | 0.62 | 0 | 0.54 | 0.51 | 0.71 | 0.61 | 0.55 | 0.26 |
| Agro-forestry areas | 0.52 | 0.62 | 0 | 0.54 | 0.51 | 0.71 | 0.61 | 0.55 | 0.26 |
| Broad-leaved forest | 0.93 | 0.77 | 0.47 | 0.67 | 0.65 | 0.85 | 0.77 | 0.66 | 0.4 |
| Coniferous forest | 0.82 | 0.76 | 0.44 | 0.63 | 0.61 | 0.84 | 0.76 | 0.68 | 0.39 |
| Mixed Forest | 0.52 | 0.62 | 0 | 0.54 | 0.51 | 0.71 | 0.61 | 0.55 | 0.26 |
| Natural grasslands | 0.86 | 0.72 | 0.52 | 0.75 | 0.6 | 0.8 | 0.71 | 0.63 | 0.42 |
| Moors and heathland | 0.81 | 0.69 | 0.51 | 0.72 | 0.6 | 0.78 | 0.71 | 0.63 | 0.39 |
| Sclerophyllous vegetation | 0.81 | 0.69 | 0.51 | 0.72 | 0.6 | 0.78 | 0.71 | 0.63 | 0.39 |
| Transitional woodland-shrub | 0.81 | 0.69 | 0.51 | 0.72 | 0.6 | 0.78 | 0.71 | 0.63 | 0.39 |
| Beaches, dunes, sands | 0.74 | 0.86 | 0.51 | 0.68 | 0.67 | 0.81 | 0.46 | 0.69 | 0.5 |
| Bare rocks | 0.55 | 0.61 | 0.35 | 0.51 | 0.46 | 0.61 | 0.57 | 0.52 | 0.3 |
| Sparsely vegetated areas | 0.55 | 0.61 | 0.35 | 0.51 | 0.46 | 0.61 | 0.57 | 0.52 | 0.3 |
| Burnt areas | 0.55 | 0.61 | 0.35 | 0.51 | 0.46 | 0.61 | 0.57 | 0.52 | 0.3 |
| Inland marshes | 0.96 | 0.79 | 0.59 | 0.8 | 0.64 | 0.84 | 0.74 | 0.69 | 0.44 |
| Salt Marshes | 0.96 | 0.79 | 0.59 | 0.8 | 0.64 | 0.84 | 0.74 | 0.69 | 0.44 |
| Salines | 0.96 | 0.79 | 0.59 | 0.8 | 0.64 | 0.84 | 0.74 | 0.69 | 0.44 |
| Intertidal flats | 0.96 | 0.79 | 0.59 | 0.8 | 0.64 | 0.84 | 0.74 | 0.69 | 0.44 |
| Water courses | 0.83 | 0.72 | 0.53 | 0.76 | 0.51 | 0.72 | 0.64 | 0.6 | 0.36 |
| Water bodies | 0.83 | 0.72 | 0.53 | 0.76 | 0.51 | 0.72 | 0.64 | 0.6 | 0.36 |
| Coastal lagoons | 0.96 | 0.79 | 0.59 | 0.8 | 0.64 | 0.84 | 0.74 | 0.69 | 0.44 |
| Estuaries | 0.96 | 0.79 | 0.59 | 0.8 | 0.64 | 0.84 | 0.74 | 0.69 | 0.44 |

*Factor 1 - the relative sensitivity of each land cover class to threats to habitats

**Table S3.2 – Habitat Quality Threats**

| **Threat** | **Max_distance (km)***** | **Weight**** | **Decay** |
| --- | --- | --- | --- |
| Buildings | 7 | 0.1 | Exponential |
| Artificial Pastures | 4 | 0.5 | Linear |
| Intensive Farming | 4 | 0.75 | Exponential |
| Railways | 3 | 0.75 | Linear |
| Motorways, Trunks and Primary roads | 5 | 0.75 | Exponential |
| Secondary and tertiary roads | 5 | 0.75 | Linear |
| Residential and service roads | 5 | 0.5 | Linear |
| Tracks and bridleways | 3 | 0.5 | Linear |

**Factor 2 - the relative impact of each threat for the ecosystems (i.e., habitat responses)

***Factor 3 - the relative distance between ecosystems and threats across the landscape, according to a distance-decay rate over the spatial scale under investigation

**Table S4 – Drought Regulation**

| **BIOPHYSICAL TABLE** | | |
| --- | --- | --- |
| **Land Cover Class** | **Plant Evapotranspiration Coefficient (Kc)** | **Rooting depth (mm)** |
| Continuous urban fabric | 0.094 | 1 |
| Discontinuous urban fabric | 0.308 | 1 |
| Industrial or commercial units | 0.204 | 1 |
| Road and rail networks and associated land | 0.187 | 1 |
| Airports | 0.292 | 1 |
| Mineral extraction sites | 0.285 | 1 |
| Dump sites | 0.195 | 1 |
| Construction sites | 0.195 | 1 |
| Green urban areas | 0.386 | 300 |
| Sport and leisure facilities | 0.43 | 100 |
| Non-irrigated arable land | 0.817 | 900 |
| Vineyards | 0.364 | 2000 |
| Fruit trees and berry plantations | 0.364 | 2000 |
| Pastures | 0.75 | 100 |
| Complex cultivation patterns | 0.75 | 100 |
| Land principally occupied by agriculture, with significant areas of natural vegetation | 0.75 | 100 |
| Broad-leaved forest | 0.805 | 2200 |
| Coniferous forest | 0.84 | 1200 |
| Mixed forest | 0.822 | 2200 |
| Natural grasslands | 0.355 | 1000 |
| Moors and heathland | 0.549 | 1000 |
| Transitional woodland-shrub | 0.84 | 1200 |
| Inland marshes | 0.395 | 2000 |
| Salt marshes | 0.174 | 2000 |
| Intertidal flats | 0.15 | 2000 |
| Water courses | 0.012 | 1 |
| Water bodies | 0.012 | 1 |
| Estuaries | 0.012 | 1 |

**Table S5 – Recreation**

| **LAND COVER SUITABILITY SCORE** | |
| --- | --- |
| **Land Cover Class** | **Score** |
| Continuous urban fabric | 0 |
| Discontinuous urban fabric | 0.1 |
| Industrial or commercial units | 0 |
| Road and rail networks and associated land | 0 |
| Port areas | 0 |
| Airports | 0 |
| Mineral extraction sites | 0 |
| Dump sites | 0 |
| Construction sites | 0 |
| Green urban areas | 1 |
| Sport and leisure facilities | 0.1 |
| Non-irrigated arable land | 0.3 |
| Permanently irrigated land | 0.3 |
| Rice fields | 0.4 |
| Vineyards | 0.5 |
| Fruit trees and berry plantations | 0.5 |
| Olive groves | 0.5 |
| Pastures | 0.6 |
| Annual crops associated with permanent crops | 0.3 |
| Complex cultivation patterns | 0.3 |
| Land principally occupied by agriculture, with significant areas of natural vegetation | 0.6 |
| Agro-forestry areas | 0.6 |
| Broad-leaved forest | 1 |
| Coniferous forest | 0.8 |
| Mixed forest | 1 |
| Natural grasslands | 0.8 |
| Moors and heathland | 0.8 |
| Sclerophyllous vegetation | 0.8 |
| Transitional woodland-shrub | 0.8 |
| Beaches, dunes, sands | 1 |
| Bare rocks | 0.8 |
| Sparsely vegetated areas | 0.7 |
| Burnt areas | 0 |
| Inland marshes | 1 |
| Salt marshes | 1 |
| Salines | 0.8 |
| Intertidal flats | 1 |
| Water courses | 1 |
| Water bodies | 1 |
| Coastal lagoons | 1 |
| Estuaries | 0.8 |

**Table S6 – Food Provisioning**

| **LAND DEDICATED TO AGRICULTURE** | | | | | | | | | | |  | |
| --- | --- | --- | --- | --- | --- | --- | --- | --- | --- | --- | --- | --- |
| **Land Cover Class** | **Area 1990 (ha)** | **%** | **Area 2000 (ha)** | **%** | **Area 2006 (ha)** | **%** | **Area 2012 (ha)** | **%** | **Area 2018 (ha)** | **%** | |  |
| Non-irrigated arable land | 1164370 | 26.79 | 1019531 | 23.99 | 893162 | 21.43 | 708882 | 16.61 | 673176 | 15.78 | |  |
| Permanently irrigated land | 123165 | 2.83 | 203838 | 4.80 | 225371 | 5.41 | 242621 | 5.68 | 259686 | 6.09 | |  |
| Rice fields | 55838 | 1.28 | 54406 | 1.28 | 42368 | 1.02 | 43196 | 1.01 | 44267 | 1.04 | |  |
| Vineyards | 205853 | 4.74 | 222573 | 5.24 | 210788 | 5.06 | 206999 | 4.85 | 210986 | 4.94 | |  |
| Fruit trees and berry plantations | 95248 | 2.19 | 100558 | 2.37 | 79494 | 1.91 | 84481 | 1.98 | 93562 | 2.19 | |  |
| Olive groves | 278288 | 6.40 | 262960 | 6.19 | 282996 | 6.79 | 330279 | 7.74 | 347798 | 8.15 | |  |
| Pastures | 53095 | 1.22 | 42078 | 0.99 | 80777 | 1.94 | 234949 | 5.50 | 228572 | 5.36 | |  |
| Annual crops associated with permanent crops | 447777 | 10.30 | 405610 | 9.54 | 328857 | 7.89 | 270703 | 6.34 | 270405 | 6.34 | |  |
| Complex cultivation patterns | 636277 | 14.64 | 610040 | 14.35 | 626084 | 15.02 | 635783 | 14.90 | 634332 | 14.87 | |  |
| Land principally occupied by agriculture | 718461 | 16.53 | 699748 | 16.46 | 769664 | 18.46 | 736259 | 17.25 | 732460 | 17.17 | |  |
| Agro-forestry areas | 568091 | 13.07 | 628658 | 14.79 | 629178 | 15.09 | 774085 | 18.14 | 771848 | 18.09 | |  |
| Total | 4,346,463 |  | 4,250,000 |  | 4,168,739 |  | 4,268,237 |  | 4,267,092 |  | |  |

**Table S7 – Erosion Prevention**

| **BIOPHYSICAL TABLE** | | |
| --- | --- | --- |
| **Land Cover Class** | **Cover Management Factor** | **Support Practices** |
| Continuos urban fabric | 0.1 | 0.9178 |
| Discontinuous urban fabric | 0.06 | 0.9178 |
| Industrial or commercial units | 1 | 0.9178 |
| Road and rail networks and associated land | 1 | 0.9178 |
| Port areas | 0.25 | 0.9178 |
| Airports | 0.25 | 0.9178 |
| Mineral extraction sites | 1 | 0.9178 |
| Dump Sites | 0.9 | 0.9178 |
| Construction Sites | 0.2 | 0.9178 |
| Green urban areas | 0.003 | 0.9178 |
| Sport and Leisure facilities | 0.06 | 0.9178 |
| Non-irrigated arable land | 0.46 | 0.9178 |
| Permanently irrigated land | 0.36 | 0.9178 |
| Rice Fields | 0.15 | 0.9178 |
| Vineyards | 0.4 | 0.9178 |
| Fruit trees and berry plantations | 0.3 | 0.9178 |
| Olive groves | 0.3 | 0.9178 |
| Pastures | 0.15 | 0.9178 |
| Annual crops associated with permanent crops | 0.35 | 0.9178 |
| Complex cultivation patterns | 0.2 | 0.9178 |
| Land principally occupied by agriculture, with significant areas of natural vegetation | 0.2 | 0.9178 |
| Agro-forestry areas | 0.13 | 0.9178 |
| Broad-leaved forest | 0.003 | 0.9178 |
| Coniferous forest | 0.003 | 0.9178 |
| Mixed Forest | 0.003 | 0.9178 |
| Natural grasslands | 0.08 | 0.9178 |
| Moors and heathland | 0.1 | 0.9178 |
| Sclerophyllous vegetation | 0.1 | 0.9178 |
| Transitional woodland-shrub | 0.05 | 0.9178 |
| Beaches, dunes, sands | 0 | 0.9178 |
| Bare rocks | 0 | 0.9178 |
| Sparsely vegetated areas | 0.45 | 0.9178 |
| Burnt areas | 0.55 | 0.9178 |
| Inland marshes | 0 | 0.9178 |
| Salt Marshes | 0 | 0.9178 |
| Salines | 0 | 0.9178 |
| Intertidal flats | 0 | 0.9178 |
| Water courses | 0 | 0.9178 |
| Water bodies | 0 | 0.9178 |
| Coastal lagoons | 0 | 0.9178 |
| Estuaries | 0 | 0.9178 |

**Table S8 – Pollination**

| **BIOPHYSICAL TABLE** | | | |
| --- | --- | --- | --- |
| **Land Cover Class** | **Nesting Resourses** | **Floral Resourses** | **Pollination Suitability Index** |
| Riparian scrubland | 0.8 | 0.9 | 0.83 |
| Broad-leaved forest | 0.8 | 0.9 | 0.83 |
| Natural grassland | 0.8 | 1 | 0.81 |
| Moors and heathland | 0.8 | 1 | 0.81 |
| Sclerophyllous vegetations | 0.8 | 1 | 0.81 |
| Transitional woodland scrub | 0.8 | 1 | 0.81 |
| Riparian forest | 0.8 | 0.5 | 0.78 |
| Fruit trees and berry plantations | 0.4 | 0.9 | 0.6 |
| Olive groves | 0.5 | 0.4 | 0.6 |
| Mixed forest | 0.8 | 0.6 | 0.55 |
| Sparsely vegetated areas | 0.7 | 0.35 | 0.52 |
| Inland marshes | 0.3 | 0.75 | 0.52 |
| Salt marshes | 0.3 | 0.55 | 0.52 |
| Coniferous forest | 0.8 | 0.3 | 0.49 |
| Annual crops associated with permanent crops | 0.4 | 0.5 | 0.47 |
| Complex cultivation patterns | 0.4 | 0.4 | 0.47 |
| Land principally occupied by agriculture | 0.7 | 0.75 | 0.47 |
| Agro-forestry areas | 1 | 0.5 | 0.47 |
| Non-irrigated arable land | 0.2 | 0.2 | 0.39 |
| Permanently irrigated land | 0.2 | 0.05 | 0.39 |
| Rice fields | 0.2 | 0.05 | 0.39 |
| Pastures | 0.3 | 0.2 | 0.39 |
| Continuous urban fabric | 0.1 | 0.05 | 0.23 |
| Discontinuous urban fabric | 0.3 | 0.3 | 0.23 |
| Industrial or commercial units | 0.1 | 0.05 | 0.23 |
| Road and rail networks | 0.3 | 0.25 | 0.23 |
| Port areas | 0.3 | 0 | 0.23 |
| Airports | 0.3 | 0 | 0.23 |
| Mineral extraction sites | 0.3 | 0.05 | 0.23 |
| Dump sites | 0.05 | 0 | 0.23 |
| Green urban areas | 0.3 | 0.25 | 0.23 |
| Sport and leisure facilities | 0.3 | 0.05 | 0.23 |
| Vineyards | 0.4 | 0.6 | 0.2 |
| Burnt areas | 0.3 | 0.2 | 0.13 |

**Table S9.**Ecosystem service indicators and their weights (wi) of relative importance measured from 0-1. ^45^.

| **Ecosystem service indicator** | **Weight (*w_i_*)** |
| --- | --- |
| Climate Regulation | 0.14 |
| Water Purification | 0.15 |
| Habitat Quality | 0.16 |
| Drought Regulation | 0.17 |
| Recreation | 0.04 |
| Food Provisioning | 0.11 |
| Erosion Prevention | 0.09 |
| Pollination | 0.18 |

**References**

1. Copernicus, CORINE Land Cover. https://land.copernicus.eu/ (2018).

2. Gómez-Baggethun, E. & Barton, D. N. Classifying and valuing ecosystem services for urban planning. *Ecol. Econ.* **86**, 235–245 (2013).

3. Sharp, R. et al. InVEST 3.10.2 User’s Guide. The Natural Capital Project, Stanford University, University of Minnesota, The Nature Conservancy, World Wildlife Fund (2020).

4. Canaveira, P., Maciel, H., Pereira, T., Pina, A. & Seabra, T. Portuguese National Inventory Report on Greenhouse Gases, 1990-2011. (2013).

5. Cunha, J., Campos, F. S., David, J., Padmanaban, R. & Cabral, P. Carbon sequestration scenarios in Portugal: which way to go forward? *Environ. Monit. Assess.* **193**, 547 (2021).

6. Correia, A. C. et al. Carbon sink strength of a Mediterranean cork oak understorey: how do semi-deciduous and evergreen shrubs face summer drought? Journal of Vegetation *Science* **25**, 411–426 (2014).

7. Almagro, M., López, J., Boix-Fayos, C., Albaladejo, J. & Martínez-Mena, M. Belowground carbon allocation patterns in a dry Mediterranean ecosystem: A comparison of two models. *Soil Biol. Biochem.* **42**, 1549–1557 (2010).

8. Nieto, O. M., Castro, J. & Fernández-Ondoño, E. Conventional tillage versus cover crops in relation to carbon fixation in Mediterranean olive cultivation. *Plant Soil.* **365**, 321–335 (2013).

9. Muñoz-Rojas, M., de la Rosa, D., Zavala, L. M., Jordán, A. & Anaya-Romero, M. Changes in land cover and vegetation carbon stocks in Andalusia, Southern Spain (1956–2007). *Sci. Total Environ.* **409**, 2796–2806 (2011).

10. Lozano-García, B. & Parras-Alcántara, L. Short-term effects of olive mill by-products on soil organic carbon, total N, C:N ratio and stratification ratios in a Mediterranean olive grove. *Agric. Ecosyst. Environ.* **165**, 68–73 (2013).

11. Eggleston, H. S., Buendia, L., Miwa, K., Ngara, T. & Tanabe, K. IPCC Guidelines for National Greenhouse Gas Inventories. 2006 IPCC Guidelines for National Greenhouse Gas Inventories https://www.ipcc-nggip.iges.or.jp/public/2006gl/index.html (2006).

12. Kanaya, G., Suzuki, T. & Kikuchi, E. Impacts of the 2011 tsunami on sediment characteristics and macrozoobenthic assemblages in a shallow eutrophic lagoon, Sendai Bay, Japan. *PLoS One* **10**, e0135125 (2015).

13. European Environment Agency. European Digital Elevation Model (EU-DEM), version 1.1. Copernicus Programme (2016).

14. Fick, S. E. & Hijmans, R. J. WorldClim 2: new 1‐km spatial resolution climate surfaces for global land areas. *Int. J. Climatol.* **37**, 4302–4315 (2017).

15. SNIG. Sistema Nacional de Informação Geográfica. http://snig.dgterritorio.pt/ (2021).

16. Deletraz, G. & Dabos, P. Modélisation statistique de la pollution azotée en proximité d’un axe routier et évaluation des incidences sur l’environnement - Application au site de Biriatou (A63 – Pyrénées-Atlantiques). in 63–93 (Laboratoire THEMA, Université de Franche-Comté, INRETS, CERTU, Editions Paradigme, 2001).

17. Foy, R. & Girvan, J. An evaluation of nitrogen sources and inputs to tidal waters in Northern Ireland(2004).

18. Jeje, Y. Export Coefficients for Total Phosphorus, Total Nitrogen and Total Suspended Solids in the Southern Alberta Region: A Review of Literature(2006).

19. Jordan, C., McGuckin, S. O. & Smith, R. V. Increased predicted losses of phosphorus to surface waters from soils with high Olsen-P concentrations. *Soil Use Manag.* **16**, 27–35 (2000).

20. Kelsey, P. & Hall, J. Nutrient-Export Modelling of the Leschenault Catchment. Water Science Technical series, Department of Water, Australia (2010).

21. Leh, M. D. K., Matlock, M. D., Cummings, E. C. & Nalley, L. L. Quantifying and mapping multiple ecosystem services change in West Africa. *Agric. Ecosyst. Environ.* ***165***, 6–18 (2013).

22. Matias, N.-G. & Johnes, P. J. Catchment Phosphorous Losses: An Export Coefficient Modelling Approach with Scenario Analysis for Water Management. *Water Resour. Manag.* **26**, 1041–1064 (2011).

23. Payraudeau, S., Tournoud, M., Cernesson, F. & Picot, B. Modélisation de la charge annuelle en azote et phosphore par analyse spatiale d’informations topographiques et d’occupation des sols - Cas d’un petit bassin versant méditerranéen. Revue EAT thématique 27–35 (2002).

24. Reckhow, K. H., Beaulac, M. N. & Simpson, J. T. Modeling Phosphorus Loading and Lake Response Under Uncertainty: A Manual and Compilation of Export Coefficients. (United States Environmental Protection Agency, Washin, 1980).

25. Wochna, A., Lange, K. & Urbanski, J. Wochna A., Lange K. and Urbanski J., 2011. The influence of land cover change during sixty years on non-point source phosphorus loads to Gulf of Gdansk. in Proceedings of the 11th International Coastal Symposium 1820–1824 (Szczecin, Poland, 2011).

26. Chaplin-Kramer, R. et al. Global modeling of nature’s contributions to people. *Science* **366**, 255–258 (2019).

27. Panagos, P., Van Liedekerke, M., Jones, A. & Montanarella, L. European Soil Data Centre: Response to European policy support and public data requirements. *Land Use Policy* **29**, 329–338 (2012).

28. Trabucco, A. & Zomer, R. J. Global Aridity Index and Potential Evapotranspiration (ET0) Climate Database v2. CGIAR Consortium for Spatial Information (CGIAR-CSI) 10 (2018) doi:10.6084/m9.figshare.7504448.v3.

29. Almeida, B. & Cabral, P. Water Yield Modelling, Sensitivity Analysis and Validation: A Study for Portugal. *ISPRS Int. J. Geoinf.* **10**, 494 (2021).

30. Vallecillo, S., La Notte, A., Zulian, G., Ferrini, S. & Maes, J. Ecosystem services accounts: Valuing the actual flow of nature-based recreation from ecosystems to people. *Ecol Model.* **392**, 196–211 (2019).

31. Zulian, G., Paracchini, M.-L., Maes, J. & Liquete Garcia, M. D. C. ESTIMAP: Ecosystem services mapping at European scale European Commission (2013). https://doi.org/10.2788/64713 (2013).

32. UNEP-WCMC & IUCN. Protected Planet: The World Database on Protected Areas (WDPA) and World Database on Other Effective Area-based Conservation Measures (WD-OECM). https://www.protectedplanet.net (2021).

33. ESRI. ArcGIS Pro 3.2. https://www.esri.com/en-us/arcgis/products/arcgis-pro/overview.

34. Cabral, P., Feger, C. C., Levrel, H., Chambolle, M. M. & Basque, D. Assessing the impact of land-cover changes on ecosystem services: A first step toward integrative planning in Bordeaux, France. *Ecosyst. Serv.* **22**, 318–327 (2016).

35. Panagos, P. et al. Global rainfall erosivity assessment based on high-temporal resolution rainfall records, 1–12 (2017). https://doi.org/10.1038/s41598-017-04282-8.

36. Panagos, P., Meusburger, K., Ballabio, C., Borrelli, P. & Alewell, C. Soil erodibility in Europe: A high-resolution dataset based on LUCAS. *Sci. Total Environ.* **479**–480, 189–200 (2014).

37. Marques, S. M., Campos, F. S., David, J. & Cabral, P. Modelling Sediment Retention Services and Soil Erosion Changes in Portugal: A Spatio-Temporal Approach. *ISPRS Int. J. Geoinf.* **10**, 262 (2021).

38. Panagos, P. et al. Estimating the soil erosion cover-management factor at the European scale. *Land Use Policy* **48**, 38–50 (2015).

39. Renard, K. G., Foster, G. R., Weesies, G. A., McCool, D. K. & Yoder, D. C. Predicting soil erosion by water: a guide to conservation planning with the Revised Universal Soil Loss Equation (RUSLE). *USDA Agriculture Handbook* **703**, 404 (1997).

40. Vallecillo, S. et al. Ecosystem services accounting: Part I - Outdoor recreation and crop pollination , EUR 29024 EN, Publications Office of the European Union, Luxembourg (2018). https://doi.org/ 10.2760/619793

41. Zulian, G., Maes, J. & Paracchini, M. Linking Land Cover Data and Crop Yields for Mapping and Assessment of Pollination Services in Europe. *Land* **2**, 472–492 (2013).

42. Greenleaf, S. S., Williams, N. M., Winfree, R. & Kremen, C. Bee foraging ranges and their relationship to body size. *Oecologia* **153**, 589–596 (2007).

43. Gathmann, A. & Tscharntke, T. Foraging ranges of solitary bees. *J. Anim. Ecol.* **71**, 757–764 (2002).

44. Cabral, P., Campos, F. S., David, J. & Caser, U. Disentangling ecosystem services perception by stakeholders: An integrative assessment based on land cover. *Ecol. Indic.* **126**, 107660 (2021).
